# Crystalline Germanium(I) and Tin(I) Centered Radical Anions

**DOI:** 10.1002/anie.202201248

**Published:** 2022-03-23

**Authors:** Li Feng Lim, Martyna Judd, Petra Vasko, Michael G. Gardiner, Dimitrios A. Pantazis, Nicholas Cox, Jamie Hicks

**Affiliations:** ^1^ Research School of Chemistry Australian National University Acton ACT, 2601 Australia; ^2^ Department of Chemistry University of Helsinki P.O. Box 55 00014 Helsinki Finland; ^3^ Max-Planck Institut für Kohlenforschung Kaiser-Wilhelm-Platz 1 45470 Mülheim an der Ruhr Germany

**Keywords:** EPR Spectroscopy, Group 14 Elements, Main Group Elements, Radical Ions

## Abstract

An isostructural series of heavy Group 14 E(I) radical anions (Ge, Sn, Pb), stabilized by a bulky xanthene‐based diamido ligand are reported. The radical anions were synthesised by the one‐electron reduction of their corresponding E(II) precursor complexes with sodium naphthalenide in THF, yielding the radical anions as charge‐separated sodium salts. The series of main group radicals have been comprehensively characterized by EPR spectroscopy, X‐ray crystallography and DFT analysis, which reveal that in all cases, the spin density of the unpaired electron almost exclusively resides in a *p*‐orbital of π symmetry located on the Group 14 center.

## Graphical abstract

An isostructural series of heavy Group 14 E(I) radical anions (Ge, Sn, Pb), stabilized by a bulky diamido ligand, is reported. Comprehensive characterization of these species was achieved by using EPR spectroscopy, X‐ray crystallography and DFT analysis. The results obtained reveal that in all cases, the spin density of the unpaired electron resides, almost exclusively, in a *p*‐orbital of π symmetry located on the metal center itself.

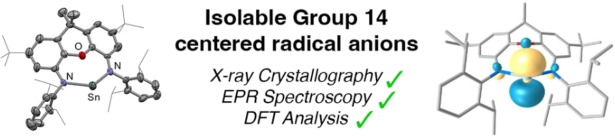

Open‐shell systems are a common occurrence in many areas of chemistry, including that of transition metal chemistry,^[1]^ organic chemistry,^[2]^ enzymatic catalysis^[3]^ and polymerization^[4]^ amongst others.^[5]^ In contrast, open‐shell systems in main group chemistry are far less common—complexes bearing unpaired electrons typically rapidly disproportionate, oligomerize or decompose via various other routes.^[6]^ Nevertheless, over the last few decades, considerable progress has been made. By carefully tailoring the steric and electronic properties of the associated ligands, several long‐lived main group centered radical species have been detected and, in some cases, even isolated.[^6^, ^7^]

In specific reference to the Group 14 elements, the vast majority of the work here has been performed on the “Gomberg‐type” radicals, leading from the pioneering work of Gomberg in 1900.^[8]^ These are trivalent neutral systems of the type [R_3_E]⋅ (E=Group 14 element, R=alkyl, aryl, amido, silyl substituent), where the Group 14 element formally occupies the +III oxidation state, in most cases.[^6^, ^7b^, ^7c^] These systems have considerably broadened our knowledge on organic and main group centered‐radicals, whilst also been shown to exhibit some interesting redox properties. However, in terms of being utilized in useful/interesting chemical transformations, reports are limited.[^6^, ^7b^, ^7c^] The low oxidation Group 14 radicals on the other hand (i.e. those in the formal oxidation state +I), have much more potential here. This can easily be rationalized by simply looking at their closely related E(I) closed‐shell counterparts (e.g. digermynes, distannynes), which have shown to be highly effective at small molecule activation, in many cases mimicking reactivity closely associated with that of the transition metals.^[9]^ That said, studies involving Group 14 E(I) radical complexes, especially those involving the heavier elements Ge, Sn and Pb are rare.[^10^, ^11^, ^12^, ^13^, ^14^, ^15^] In 1995, Egorov, Gaspar and co‐workers reported the in situ generation of the first anionic E(I) heavy Group 14 radical anions [{(Me_3_Si)_2_CH}_2_E]^.−^ (E=Ge, Sn) **I** (Figure 1) by the one‐electron reduction of the corresponding neutral tetrylene precursor complexes.^[11]^ In 2011, Kaupp, Murphy, Driess, Jones and co‐workers successfully isolated the first heavy Group 14 E(I) radical, [(Nacnac)Ge]⋅ **II** and characterized the complex in the solid state.^[12]^ More recently, Stalke, Frenking, Kiam, Roesky and co‐workers successfully isolated an acyclic Ge(I) radical, using a combination of an amido ligand and a cyclic(alkyl)(amino)carbene (cAAC) **III**.^[13]^ However, the team found a considerable contribution of a resonance form where the unpaired electron was localized on the cAAC ligand. In 2018, Power and co‐workers demonstrated the power of these systems in bond‐activation, by successfully achieving C−H activation of toluene with a Sn(I) radical‐containing system, generated in situ via photolysis or thermolysis of a bulky diarylstannylene **IV**.^[14]^ The same group more recently reported that the same Sn(I) radical can be generated by photolysis or thermolysis of a distannyne.^[15]^ In this work, we report the first isostructural series of Group 14 E(I) radical anions from Ge to Pb. Through comprehensive characterization, we show that the unpaired electron resides almost exclusively on the Group 14 center in all 3 homologues.

**Figure 1:**
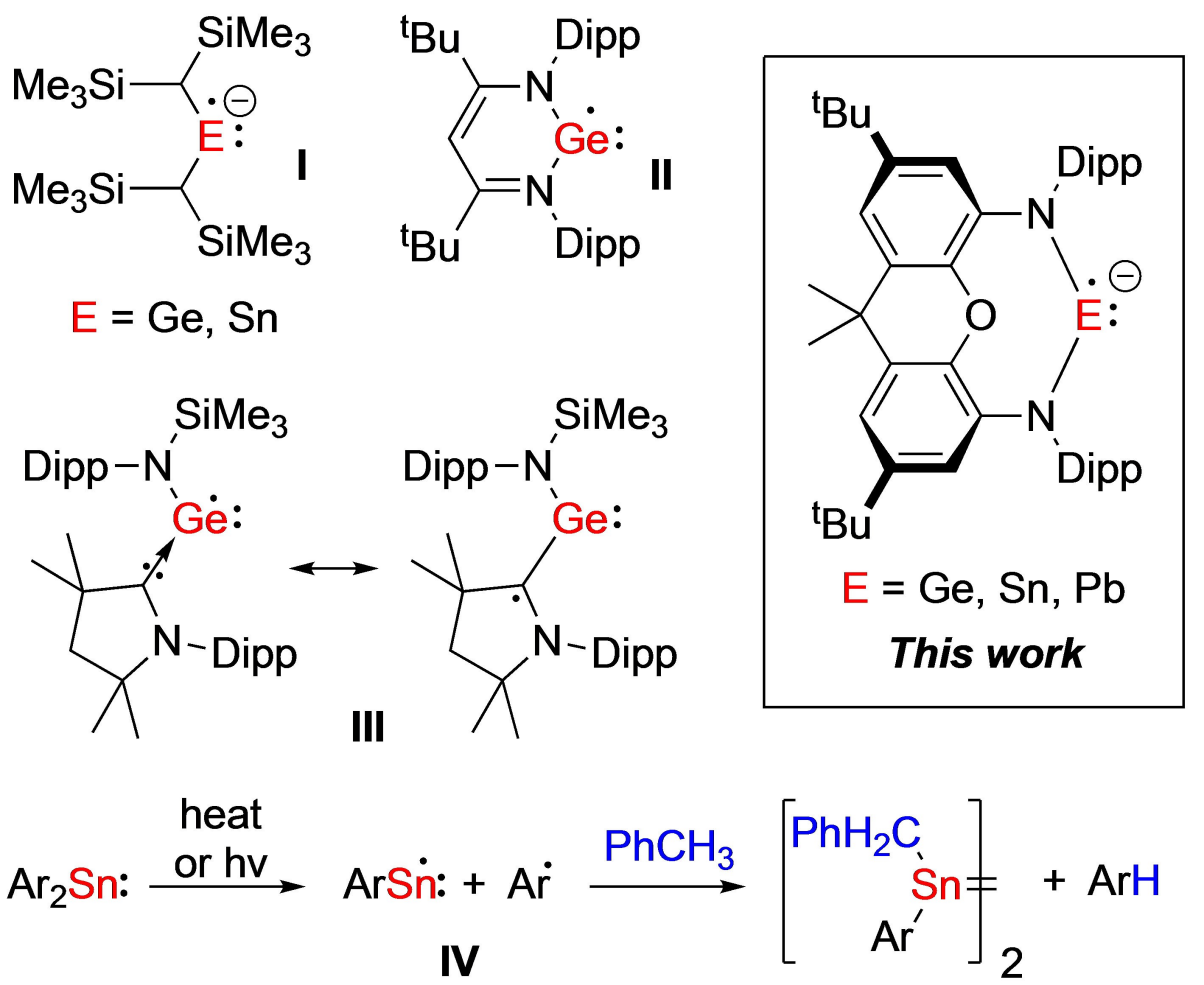
Heavy monometallic Group 14 E(I) radical complexes.

In recent work, we have been successful in utilizing the bulky xanthene‐based diamido ligand **NON** (**NON**=4,5‐bis(2,6‐diisopropylanilido)−2,7‐di‐*tert*‐butyl‐9,9‐dimethyl‐xanthene) in the stabilisation of a range of highly reactive main group species,^[16]^ and hypothesized that this ligand may also be suited to stabilize heavy Group 14 E(I) radical anions. In addition, the neutral E(II) tetrylene complexes (**NON**)E: (E=Ge, **1‐Ge**; Sn, **1‐Sn**, Pb, **1‐Pb**) bearing the **NON** ligand have recently been reported by Breher,^[17]^ which could be envisioned to serve as precursors to the radical anions, with the LUMO being the *p_z_* orbital located on the Group 14 center (see Supporting Information). The neutral tetrylene complexes used in this work were synthesized via a modified procedure to that reported,^[17]^ via simple one‐pot salt metathesis reactions between the dilithium salt of the ligand, Li_2_(**NON**), and the corresponding Group 14 dihalide salt. These reactions led to three tetrylene complexes **1‐Ge**, **1**‐**Sn** and **1‐Pb**) being isolated in high crystalline yields (68—86 %, see Supporting Information for further details).

With the three tetrylene complexes in hand, one‐electron reductions of all three complexes were investigated. Various hydrocarbon and ether solutions of **1‐Ge**, **1‐Sn**, and **1‐Pb** were exposed to a range of reducing reagents (e.g. Na, K, KC_8_) however, in all cases, this led to ‘over‐reduction’ of the Group 14 complex to give deposition of group 14 metal in the elemental form. More success was found when a THF solution of sodium naphthalenide (1 equiv) was added dropwise to a THF solution of **1‐Ge**, **1‐Sn** or **1‐Pb** at low temperature (−78 °C for Ge and Sn; −95 °C for Pb). Immediately on addition, this gave deep red solutions of the corresponding radical anions [(**NON**)E:]^.−^ (E=Ge, **2‐Ge**; Sn, **2‐Sn**, Pb, **2‐Pb**) as their sodium salts (Scheme 1).

**Scheme 1:**
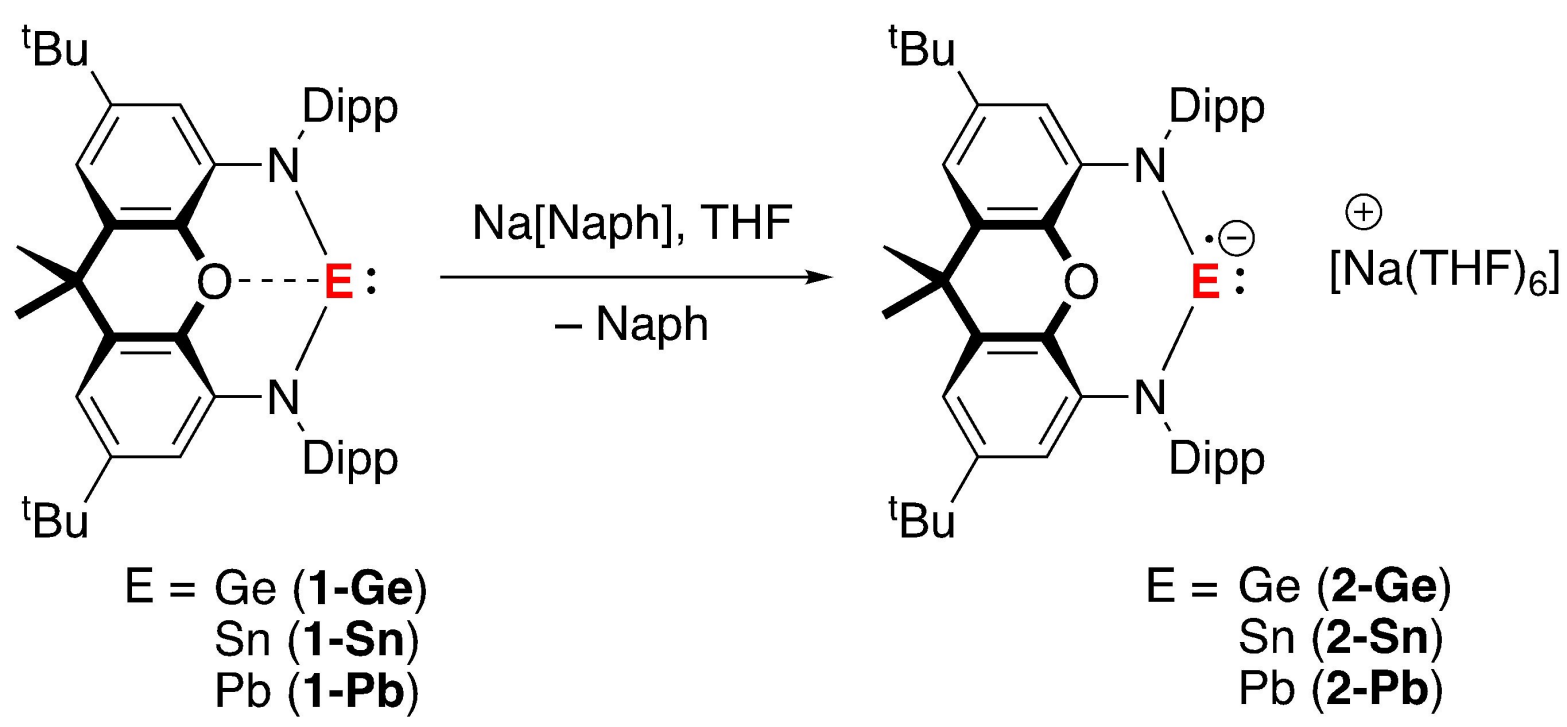
Synthesis of the radical anions **2‐Ge**, **2‐Sn** and **2‐Pb**.

All three radical anions (**2‐Ge**, **2‐Sn**, **2‐Pb**) were found to be highly thermally sensitive, both in solution and in the solid state. Decomposition of THF solutions of **2‐Ge** were found to commence at around −40 °C, as observed by an obvious solution colour change (from deep red to brown) and the precipitation of the Group 14 metal. THF solutions of **2‐Sn** on the other hand were found to be slightly more stable, up until approximately −25 °C where slow decomposition was observed (most notably by the slow formation of a tin mirror). The Pb analogue **2‐Pb** was found to be the most thermally sensitive of the three, with slow decomposition observed even at −78 °C. As such, an optimized procedure to generate **2‐Pb** was achieved by performing the reduction at −95 °C. After warming to room temperature, the three ‘decomposed’ solutions were analyzed by ^1^H NMR, which revealed an almost stoichiometric 1 : 1 ratio of the corresponding E(II) tetrylene (**1‐Ge**, **1‐Sn** or **1‐Pb**) and Na_2_(**NON**). These observations suggest decomposition of the E(I) radical anions occurs by a disproportionation process.^[6]^

As solid‐state characterization of monometallic heavy Group 14 E(I) radical complexes is limited (**II** and **III** shown in Figure 1 being the sole examples),[^12^, ^13^] we were keen to analyze the series of radical anions by X‐ray crystallography. Single crystals of **2‐Ge** and **2‐Sn** were grown by slow evaporation of their THF reaction solutions, held at low temperatures (−40 °C for **2‐Sn** and −78 °C for **2‐Ge**) over several days. This resulted in deep red crystals of **2‐Ge** and **2‐Sn** suitable for X‐ray diffraction (Figure 2).^[18]^ Unfortunately, single crystals of **2‐Pb** could not be isolated due to the compound's considerably higher thermal instability.

**Figure 2:**
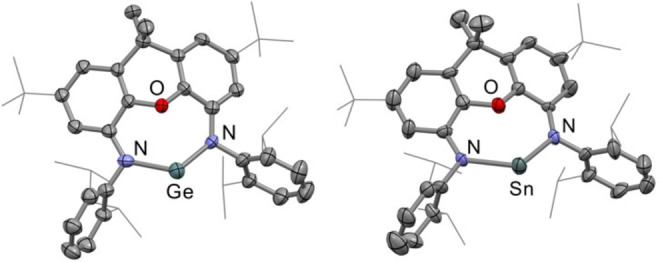
Molecular structures of **2‐Ge** (left) and **2‐Sn** (right) as determined by X‐ray crystallography. Hydrogen atoms and the [Na(THF)_6_]^+^ cations are omitted for clarity. Displacement ellipsoids set at the 50 % probability level. Key bond lengths [Å]; **2‐Ge**: Ge−N 2.089(5), 2.072(6); Ge⋅⋅⋅O 2.641(4); **2‐Sn**: Sn−N 2.260(11), 2.281(10); Sn⋅⋅⋅O 2.61(1).^[18]^

**2‐Ge** and **2‐Sn** represent the first structurally characterized monometallic Ge(I) and Sn(I) radical anions, with **2‐Sn** being the first structurally characterized Sn(I) radical of any kind. At first glance, the structures of **2‐Ge** and **2‐Sn** appear to be very similar to those of their tetrylene precursor complexes **1‐Ge** and **1‐Sn**,^[17]^ but with the obvious addition of a [Na(THF)_6_] cation in the lattice in both cases. The presence of this cation suggests that an electron has successfully been transferred onto the (**NON**)E: unit, generating a solvent separated ion pair in both cases. More subtle differences can also be observed in the bond lengths and angles around the Group 14 center. The Ge−N bond lengths in **2‐Ge** for example, are pprox.. 0.1 Å longer than those in **1‐Ge** (2.089(5)/2.072(6) Å in **2‐Ge** cf. 1.983(3)/1.973(3) Å in **1‐Ge**)^[17]^ and the Ge⋅⋅⋅O distance in **2‐Ge**, a significant 0.4 Å longer than that in **1‐Ge** (2.641(4) Å in **2‐Ge** cf. 2.213(2) Å in **1‐Ge**).^[17]^ In contrast, the N−E−N angle in both radical anions does not change significantly from that in their precursor complexes (N−Ge−N 117.65(2)° **1‐Ge** cf. 117.2(2)° in **2‐Ge**; N−Sn−N 115.83(10)° **1‐Sn** cf. 119.8(4)° in **2‐Sn**).^[17]^ However, the “hinging” of the ligand's xanthene backbone has considerably decreased from **1‐Ge** to **2‐Ge** (47.8° from planar in **1‐Ge** cf. 43.9° in **2‐Ge**). Similar observations can be seen in the structure of **2‐Sn**, with an increase in the Sn−N and Sn⋅⋅⋅O distances of approximately 0.1 and 0.2 Å, respectively, compared to those in **1‐Sn** (**1‐Sn**: Sn−N 2.182(3) Å, Sn⋅⋅⋅O 2.369(2) Å; **2‐Sn**: Sn−N 2.260(11)/2.281(10) Å, Sn⋅⋅⋅O 2.61(1) Å). These observations are consistent with an increase in the Group 14 element's ionic radius, which would be a result of a formal one electron reduction from +II to +I. Similar observations have been seen in reducing **NON** Group 13 complexes from +III to +I.^[16a]^ Thus structural analysis alone suggests that the unpaired electron on both anions is located on the Group 14 center.

To gain a better understanding of the electronic structure of the radical anions, all three complexes were additionally investigated by density functional theory (DFT) and electron paramagnetic resonance (EPR) spectroscopy. DFT models of **2‐Ge**, **2‐Sn** and **2‐Pb** support the assignment of metal‐centered radicals in all cases. For all three systems, the SOMO represents an almost exclusive unhybridized *p*‐orbital (Figure 3). In addition, all bond lengths were reproduced within crystallographic error. Importantly, these models also explain the increase in the E⋅⋅⋅O distance upon the reduction, which was found to arise due to the antibonding interaction between the oxygen lone pair and the unpaired electron on the E(I) center, which both display π‐symmetry and decreases down the group. Furthermore, EPR measurements unambiguously demonstrate that **2‐Ge**, **2‐Sn** and **2‐Pb** are all metal centered radicals. Their EPR spectra (Figure 3) are best viewed as the superposition of two discrete signals, a dominant sharp signal in which the Group 14 center is NMR inactive (*I*=0) and an underlying signal in which the Group 14 center is NMR active (*I*>0). Good agreement is seen between experimental and calculated EPR parameters validating the DFT electronic structural models (see Supporting Information for further details).

**Figure 3:**
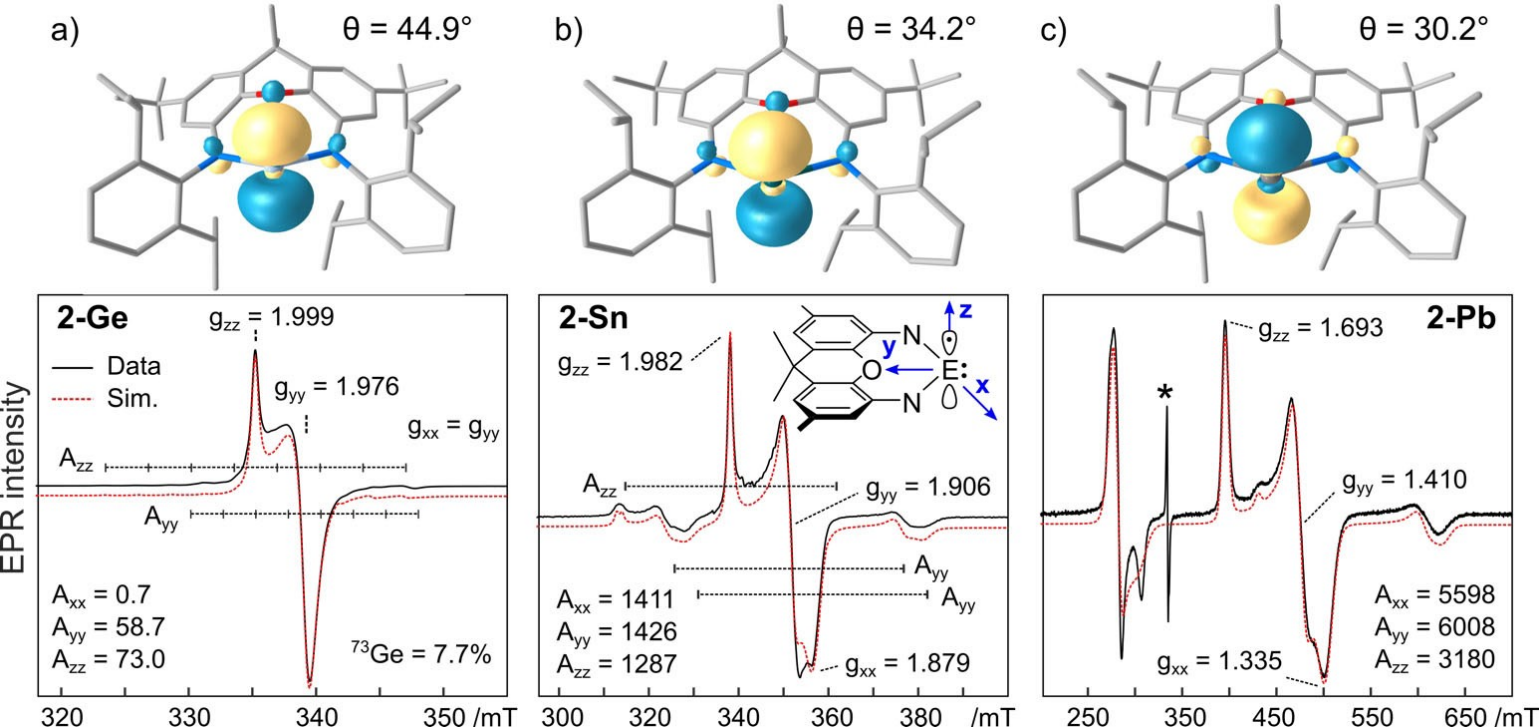
Singly occupied molecular orbitals (SOMOs) from DFT models (top) and experimental EPR spectra (bottom) of a) **2‐Ge**; b) **2‐Sn**_;_ and c) **2‐Pb**. The SOMO is shown for each complex along with the “hinging” of the ligand's backbone (θ). Spin Hamiltonian simulations, offset from the data are shown in red and all parameters listed. * indicates an unidentified trace paramagnetic impurity.

From the EPR data, two clear trends can be observed descending the group: a) the anisotropy of the *g* and hyperfine tensors increase; and b) the magnitude of the hyperfine tensor increases. These trends can be explained through decomposition of the full tensors (*A*_TOTAL_) into three individual contributions: i) spin—dipole coupling (*A*_SD_); ii) spin—orbit coupling (*A*_SO_); and iii) Fermi contact (*A*_FC_) interaction (see Supporting Information for further details). All three increase significantly, as expected descending the group, i.e. heavier elements of a group should display larger relativistic effects owing to core orbital contraction and a larger Fermi contact interaction due to the larger electronic wavefunction overlap at the nucleus.^[19]^ The larger spin—spin and spin—orbit contribution readily explains trend (a), and the larger Fermi contact term, trend (b)—specifically it leads to an increase in the isotropic hyperfine coupling (*A*_iso_). The magnitude of *A*_iso_ provides the clearest indication that the unpaired electron occupies a metal‐centered *p*‐orbital. The Fermi contact term is associated with *s*‐orbital occupancy, with a SOMO of only *s*‐character representing the theoretical maximum (*A*_max_) hyperfine coupling constant. Taking the ratio of *A*_iso_/*A*_max_ we find that for **2‐Ge** it is only 1.5 %, increasing to 3 % for **2‐Sn** and 6 % for **2‐Pb**.

Comparing the tensors recorded for the three radical anions to the handful of reported E(I) radical complexes leads to some interesting observations. The *g* and hyperfine tensors for **2‐Ge** are almost identical as those reported for the *β*‐diketiminate stabilised Ge(I) radical (**II**, Figure 1)^[12]^ which is not overly surprising considering their similar structure (see Supporting Information Table S5.4 for a side‐by‐side comparison). More interesting though, is that the isotropic hyperfine constant reported for the transient Sn(I) radical (**IV**, Figure 1) is also in good agreement with that recorded for **2‐Sn** (see Supporting Information Table S5.5).[^14^, ^15^] Although the two radical species are structurally different (e.g. 2‐coordinate vs. 1‐coordinate, N‐substituents vs. C‐substituent), neither ligand field allows significant mixing of the singly occupied 5*p* orbital with that of the 5s orbital. No Pb(I) radicals are described in current literature and therefore direct comparisons to **1‐Pb** are difficult. That said, comparisons can be made to the corresponding Pb(III) systems, in which the unpaired electron is localized in a similar unhybridized Pb‐centered 6*p* orbital.^[20]^ However, the problem with these comparisons is that all reported Pb(III) radical complexes display some sort of pyramidalization of the Pb center (i.e. not planar), which leads to larger isotropic hyperfine tensors due to greater *s*‐character of the SOMO. A such, the best comparisons here are made with the “almost planar” radical complex [(Me_3_Si)_2_EtSi)]_3_Pb⋅ reported by Klinkhammer and co‐workers,^[20a]^ which have hyperfine tensors that closely match those of **2‐Pb** in terms of both magnitude and rhombicity (see Supporting Information Table S5.6).

Corresponding double resonance measurements were also performed on all 3 radical anions, which probe the spin density found on the **NON** ligand (Figure 4). These measurements complement the above results, finding that little spin density is localized on the ligand itself, by small ^14^N and ^1^H hyperfine couplings of <5 MHz for all three radical anions (see Supporting Information for further details).

**Figure 4:**
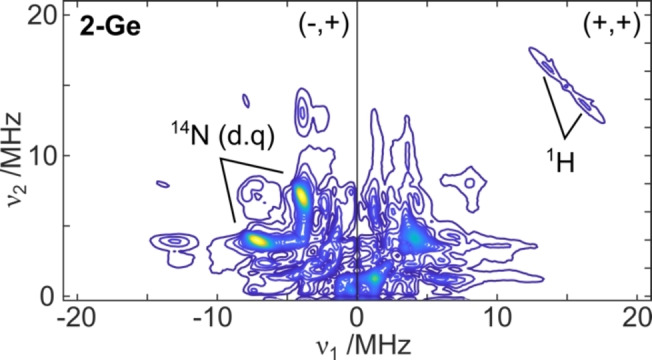
X‐band HYSCORE spectrum of **2‐Ge**. ^1^H and ^14^N signals marked are consistent with hyperfine coupling constants of the order of 4 MHz. See Supporting Information for corresponding **2‐Sn** and **2‐Pb** data.

In summary, the first series of heavy Group 14 radical anions (Ge, Sn and Pb) is reported, which have been comprehensively characterized by X‐ray crystallography, EPR spectroscopy and DFT analysis. In all characterization techniques, results were consistent in supporting the assignment of a metal‐centered radical in all anions. Even though **2‐Pb** could not be analyzed in the solid state, the work here represents the first characterization of a Pb(I) radical. Investigations into the reactivity of these open‐shell main group species are currently underway.

## Conflict of interest

The authors declare no conflict of interest.

## Supporting information

As a service to our authors and readers, this journal provides supporting information supplied by the authors. Such materials are peer reviewed and may be re‐organized for online delivery, but are not copy‐edited or typeset. Technical support issues arising from supporting information (other than missing files) should be addressed to the authors.

Supporting Information

Supporting Information

## Data Availability

The data that support the findings of this study are available in the supplementary material of this article.

## References

[1] See for example

[1a] M. Gerloch , E. C. Constable in Transition Metal Chemistry: The Valence Shell in d-Block Chemistry; Wiley-VCH, Weinheim, 1994;

[1b] R. H. Crabtree in , The Organometallic Chemistry of the Transition Metals, 7th ed., Wiley, Hoboken, 2019.

[2a] J. Clayden , N. Greeves , S. G. Warren , in Organic chemistry , 2nd ed., Oxford University Press, Oxford, 2012;

[2b] D. Griller , K. U. Ingold , Acc. Chem. Res. 1976, 9, 13–19;

[2c] P. Renaud , M. P. Sibi , in Radicals in Organic Synthesis, Wiley-VCH, Weinheim, 2008.

[3a] P. A. Frey , Annu. Rev. Biochem. 2001, 70, 121–148; 11395404 10.1146/annurev.biochem.70.1.121

[3b] W. Buckel , B. T. Golding , FEMS Microbiol. Rev. 1998, 22, 523–541.

[4] See for example

[4a] G. Odian , in Principles of Polymerization, 4th ed., Wiley-Interscience, Hoboken, 2004;

[4b] W. A. Braunecker , K. Matyjaszewski , Prog. Polym. Sci. 2007, 32, 93–146.

[5] Encyclopedia of Radicals in Chemistry, Biology and Materials (Eds.: C. Chatgilialoglu , A. Studer ), Wiley, Hoboken, 2012.

[6a] P. P. Power , Chem. Rev. 2003, 103, 789–809; 12630853 10.1021/cr020406p

[6b] P. J. Davidson , A. Hudson , M. F. Lappert , P. W. Lednor , J. Chem. Soc. Chem. Commun. 1973, 829–830;

[6c] A. Hudson , M. F. Lappert , P. W. Lednor , J. Chem. Soc. Dalton Trans. 1976, 2369–2375;

[6d] M. J. S. Gynane , M. F. Lappert , P. I. Riley , P. Rivière , M. Rivière-Baudet , J. Organomet. Chem. 1980, 202, 5–12.

[7a] C. D. Martin , M. Soleilhavoup , G. Bertrand , Chem. Sci. 2013, 4, 3020–3030; 23878717 10.1039/C3SC51174J PMC3714118

[7b] V. Y. Lee , M. Nakamoto , A. Sekiguchi , Chem. Lett. 2008, 37, 128–133;

[7c] V. Y. Lee , A. Sekiguchi , Eur. J. Inorg. Chem. 2005, 1209–1222;

[7d] G. Tan , X. Wang , Chin. J. Chem. 2018, 36, 573–586.

[8] M. Gomberg , J. Am. Chem. Soc. 1900, 22, 757–771.

[9] See for example

[9a] P. P. Power , Nature 2010, 463, 171–177; 20075912 10.1038/nature08634

[9b] P. P. Power , Organometallics 2007, 26, 4362–4372;

[9c] M. Asay , C. Jones , M. Driess , Chem. Rev. 2011, 111, 354–396; 21133370 10.1021/cr100216y

[9d] C. Cui , M. Brynda , M. M. Olmstead , P. P. Power , J. Am. Chem. Soc. 2004, 126, 6510–6511. 15161252 10.1021/ja0492182

[10a] M. M. Olmstead , L. Pu , R. S. Simons , P. P. Power , Chem. Commun. 1997, 1595–1596;

[10b] S.-P. Chia , E. Carter , H.-W. Xi , Y. Li , C.-W. So , Angew. Chem. Int. Ed. 2014, 53, 8455–8458; 10.1002/anie.201404357 24924768

[10c] S. Ishida , T. Iwamoto , M. Kira , J. Am. Chem. Soc. 2003, 125, 3212–3213; 12630866 10.1021/ja028192g

[10d] S. Inoue , M. Ichinohe , A. Sekiguchi , J. Am. Chem. Soc. 2007, 129, 6096–6097; 17441724 10.1021/ja0711314

[10e] S. Inoue , M. Ichinohe , A. Sekiguchi , Organometallics 2008, 27, 1358–1360.

[11] M. P. Egorov , O. M. Nefedov , T.-S. Lin , P. Gasper , Organometallics 1995, 14, 1539–1541.

[12] W. D. Woodul , E. Carter , R. Müller , A. F. Richards , A. Stasch , M. Kaupp , D. M. Murphy , M. Driess , C. Jones , J. Am. Chem. Soc. 2011, 133, 10074–10077. 21662245 10.1021/ja204344e

[13] M. M. Siddiqui , S. K. Sarkar , S. Sinhababu , P. N. Ruth , R. Herbst-Irmer , D. Stalke , M. Ghosh , M. Fu , L. Zhao , D. Casanova , G. Frenking , B. Schwederski , W. Kaim , H. W. Roesky , J. Am. Chem. Soc. 2019, 141, 1908–1912. 30633503 10.1021/jacs.8b13434

[14] T. Y. Lai , J. C. Fettinger , P. P. Power , J. Am. Chem. Soc. 2018, 140, 5674–5677. 29648820 10.1021/jacs.8b01878

[15] T. Y. Lai , L. Tao , R. D. Britt , P. P. Power , J. Am. Chem. Soc. 2019, 141, 12527–12530. 31345027 10.1021/jacs.9b06845

[16a] J. Hicks , P. Vasko , J. M. Goicoechea , S. Aldridge , Nature 2018, 557, 92–95; 29662211 10.1038/s41586-018-0037-y

[16b] J. Hicks , A. Mansikkamäki , P. Vasko , J. M. Goicoechea , S. Aldridge , Nat. Chem. 2019, 11, 237–241; 30664716 10.1038/s41557-018-0198-1

[16c] J. Hicks , P. Vasko , J. M. Goicoechea , S. Aldridge , J. Am. Chem. Soc. 2019, 141, 11000–11003; 31251586 10.1021/jacs.9b05925

[16d] J. S. McMullen , A. J. Edwards , J. Hicks , Dalton Trans. 2021, 50, 8685–8689; 34160514 10.1039/d1dt01532j

[16e] M. M. D. Roy , J. Hicks , A. Heilmann , A.-M. Bason , P. Vasko , J. M. Goicoechea , S. Aldridge , Angew. Chem. Int. Ed. 2021, 60, 22301–22306; 10.1002/anie.202109416 34396660

[17] F. Krämer , M. S. Luff , U. Radius , F. Weigend , F. Breher , Eur. J. Inorg. Chem. 2021, 3591–3600.

[18] Deposition Numbers 2141418 (**2-Ge**) and 2141419 (**2-Sn**) contain the supplementary crystallographic data for this paper. These data are provided free of charge by the joint Cambridge Crystallographic Data Centre and Fachinformationszentrum Karlsruhe Access Structures service. Two near identical molecules of [(**NON**)Sn:]^.−^ appear in the asymmetric unit of **2-Sn**.

[19] J. R. Morton , K. F. Preston , J. Magn. Reson. 1978, 30, 577–582.

[20a] C. Förster , K. W. Klinkhammer , B. Tumanskii , H.-J. Krüger , H. Kelm , Angew. Chem. Int. Ed. 2007, 46, 1156–1159; 10.1002/anie.200603323 17171746

[20b] D. Kurzbach , S. Yao , D. Hinderberger , K. W. Klinkhammer , Dalton Trans. 2010, 39, 6449–6459. 20532294 10.1039/c001144d

