# Supplementary material for: Crystalline Germanium(I) and Tin(I) Centered Radical Anions
Source: Angew Chem Int Ed Engl. 2022 Mar 23;61(21):e202201248. doi: 10.1002/anie.202201248 (PMC9401049; doi:10.1002/anie.202201248)
Supplement: Supplementary file 2 — Supporting Information [file ANIE-61-0-s002.pdf]

Angewandte Chemie
Eine Zeitschrift der Gesellschaft Deutscher Chemiker

Supporting Information

Crystalline Germanium(I) and Tin(I) Centered Radical Anions

L. F. Lim, M. Judd, P. Vasko, M. G. Gardiner, D. A. Pantazis, N. Cox, J. Hicks**

Supplementary Information

Table of Contents

S1) Materials and methods

S2) X-ray crystallographic studies

S3) EPR materials and methods

S4) Computational details and additional data

S5) Tabulated EPR and DFT data. Comparison to literature

S6) Additional pulse EPR data

S7) Cartesian coordinates of optimized structures for **2-Ge**, **2-Sn** and **2-Pb**

S8) References

S1) Materials and methods

Synthetic and characterizing data for all compounds

General considerations. All manipulations were carried out using standard Schlenk line or dry-box techniques under an atmosphere of argon. Toluene was dried by passing through a column of activated alumina. THF was dried by refluxing over sodium and benzophenone before distillation. Benzene was dried by refluxing over potassium before distillation. All solvents were stored in Teflon valved ampoules over a sodium mirror and sparged with argon prior to use. NMR spectra were measured in benzene-d_6, which was dried by refluxing over potassium, distilled under reduced pressure and stored in a Teflon valve ampoule over 4 Å molecular sieves. NMR samples were prepared under argon in 5 mm Norell Select Series NMR Tube fitted with J. Young Teflon valves. NMR spectra were recorded on a Bruker Avance 400 MHz spectrometer. ^{1}H and ^{13}C{^{1}H} spectra were referenced internally to residual protio-solvent (^{1}H) or solvent (^{13}C) resonances and are reported relative to tetramethylsilane (δ = 0 ppm). H_2(**NON**) was prepared by the literature method.[S1] **1-Ge**, **1-Sn** and **1-Pb** were prepared by a modified procedure to that previously reported,[S2] details below. All other reagents were used as received. Due to the room temperature instability of **2-Ge**, **2-Sn** and **2-Pb** in the solid state, microanalysis could not be performed on these compounds.

Preparation of 1-Ge: To solution of H_2(**NON**) (2.00 g, 2.97 mmol) in toluene (15 mL) was added a solution of 1.6 M ^{n}BuLi in hexane (3.9 mL, 6.2 mmol) dropwise at –78 °C. The reaction mixture was allowed to warm to room temperature where it was stirred for a further 2 h. This mixture was added to a solution of $GeCl_2$(dioxane) (0.757 g, 3.27 mmol) in toluene (5 mL) dropwise at –78 °C, before the reaction mixture was slowly warmed to room temperature and stirred overnight. The reaction mixture was filtered and the orange-brown filtrate concentrated *in vacuo* (*ca.* 5 mL), which was left to stand at room temperature overnight to give **1-Ge** as orange-brown crystals. Yield 1.51 g (2.03 mmol, 68%). Characterizing data was consistent with that previously reported.[S2]

Preparation of 1-Sn: To a solution of H_2(**NON**) (2.00 g, 2.97 mmol) in THF (10 mL) was added a solution of 1.6 M ^{n}BuLi in hexane (3.9 mL, 6.2 mmol) dropwise at –78 °C. The reaction mixture was allowed to warm to room temperature where it was stirred for a further 2 h. This mixture was added to a solution of $SnCl_2$ (0.620 g, 3.27 mmol) in THF (10 mL) dropwise at –78 °C, before the reaction mixture was slowly warmed to room temperature and stirred overnight. Volatiles were removed from the yellow solution *in vacuo* and the residue extracted into benzene (20 mL) and filtered. The filtrate was concentrated in vacuo (*ca.* 5 mL) and left to stand at room temperature overnight to give **1-Sn** as bright orange crystals. Yield 1.67 g (2.11 mmol, 71%) over three crystallizations. Characterizing data was consistent with that previously reported.[S2]

Preparation of 1-Pb: To a solution of H_2(**NON**) (2.00 g, 2.97 mmol) in THF (10 mL) was added a solution of 1.6 M ^{n}BuLi in hexane (4.0 ml, 6.4 mmol) dropwise at –78 °C. The reaction mixture was allowed to warm to room

temperature where it was stirred for a further 2 h. This mixture was added to a solution of $PbCl_2$ (0.826 g, 2.97 mmol) in THF (10 mL) dropwise at –78 °C, before the reaction mixture was slowly warmed to room temperature and stirred overnight. Volatiles from the dark orange solution were removed *in vacuo* to give a purple residue, which was extracted into benzene (20 mL), filtered and the filtrate concentrated *in vacuo* to *ca.* 5 mL. The mixture was left to stand at room temperature overnight to give **1-Pb** as dark purple crystals. Yield 2.26 g (2.57 mmol, 86%). Characterizing data was consistent with that previously reported.[S2]

Preparation of 2-Ge: A solution of sodium naphthalenide was freshly prepared by transferring a solution of naphthalene (37.9 mg, 0.296 mmol) in THF (3 mL) into a Schlenk flask containing sodium metal (20 mg, 0.87 mmol) at room temperature. The reaction mixture was stirred for 12 h at room temperature, producing a deep green solution. This solution was added dropwise to a solution of **1-Ge** (200 mg, 0.269 mmol) in THF (2 mL) at –78 °C, forming a deep red/purple solution. This solution was used directly for the EPR characterization. This solution was found to be extremely, air, moisture and temperature sensitive – decomposing rapidly above –40 °C. As such, single crystals of **2-Ge** were grown by slow evaporation of this solution over the course of several days, with the temperature of the solution maintained at –78 °C. The slow evaporation was achieved by putting the reaction vessel under high vacuum (approx. 5×10^{-5} mBar) using a turbopump. This gave **2-Ge** as thermally sensitive dark red crystals in approximately 40% yield (0.130 g).

Due to the high thermal sensitivity of **2-Ge** both in solution and the solid state, the compound could not be characterized by common techniques such as elemental analysis and mass spectrometry. That said, the compound has been rigorously characterized by EPR spectroscopy, X-ray crystallography and DFT analysis.

Preparation of 2-Sn: A solution of sodium naphthalenide was freshly prepared by transferring a solution of naphthalene (35.6 mg, 0.278 mmol) in THF (3 mL) into a Schlenk flask containing sodium metal (20 mg, 0.87 mmol) at room temperature. The reaction mixture was stirred for 12 h at room temperature, producing a deep green solution. This solution was added dropwise to a solution of **1-Sn** (200 mg, 0.253 mmol) in THF (2 mL) at –78 °C, forming a dark orange solution. This solution was used directly for the EPR characterization. This solution was found to be extremely, air, moisture and temperature sensitive – decomposing above –25 °C. As such, single crystals of **2-Sn** were grown by slow evaporation of this solution over the course of several hours, with the temperature of the solution maintained at –40 °C. The slow evaporation was achieved by putting the reaction vessel under high vacuum (approx. 5×10^{-2} mBar) using a standard Edwards RV3 vacuum pump. This gave **2-Sn** as thermally sensitive dark red crystals in approximately 60% yield (0.190 g).

Due to the high thermal sensitivity of **2-Sn** both in solution and the solid state, the compound could not be characterized by common techniques such as elemental analysis and mass spectrometry. That said, the compound has been rigorously characterized by EPR spectroscopy, X-ray crystallography and DFT analysis.

Preparation of 2-Pb: A solution of sodium naphthalenide was freshly prepared by transferring a solution of naphthalene (29.2 mg, 0.228 mmol) in THF (3 mL) into a Schlenk flask containing sodium metal (20 mg, 0.87 mmol) at room temperature. The reaction mixture was stirred for 12 h at room temperature, producing a deep green solution. This solution was added dropwise to a solution of **1-Pb** (200 mg, 0.228 mmol) in THF (2 mL) at –95 °C, forming a dark orange solution. This solution was used directly for the EPR characterization. This solution was found to be extremely, air, moisture and temperature sensitive – decomposing slowly even at –78 °C. Unfortunately, all attempts to crystallize this radical anion failed, likely due to the compound's extremely unstable nature.

Due to the high thermal sensitivity of **2-Pb** both in solution and the solid state, the compound could not be characterized by common techniques such as elemental analysis and mass spectrometry. That said, the compound has been rigorously characterized by EPR spectroscopy and DFT analysis.

S2) X-ray crystallographic studies

Single-crystal X-ray diffraction data were collected using a Rigaku Supernova dual-source diffractometer. Crystals of both **2-Ge** and **2-Sn** were both found to be highly temperature sensitive, instantly decomposing in room temperature oil. As such, crystals of both were selected at low temperature (–40 °C) under a 2:1 mixture of n-hexane and Paratone-N oil, using a mCHILL cold mounting device.[S3] Crystals were mounted on Micromount loops, which were quickly plunged into liquid nitrogen before being transferred onto to goniometer of the diffractometer, which was cooled by an Oxford Cryosystems open flow N_2 cooling device.[S4] Data were collected at 150 K using mirror monochromated Cu Kα (λ = 1.5418 Å) radiation. Data collected were processed using the CrysAlisPro package, including unit cell parameter refinement and inter-frame scaling (which was carried out using SCALE3 ABSPACK within CrysAlisPro).[S5] Equivalent reflections were merged and diffraction patterns processed with the CrysAlisPro suite.[S5] Structures were subsequently solved using SHELXT-2018[S6] and refined on F^2 using SHELXL-2018[S7] in combination with the graphical interface Olex2.[S8]

Finalised CIFs for both structures have been deposited at the Cambridge Crystallographic Data Centre (2141418-2141419). These can be obtained free-of-charge via www.ccdc.cam.ac.uk/data_request/cif, by emailing data_request@ccdc.cam.ac.uk, or by contacting The Cambridge Crystallographic Data Centre, 12 Union Road, Cambridge CB2 1EZ, UK; fax: +44 1223 336033.

Table S2.1. Crystallographic and refinement parameters for the structures of compounds **2-Ge** and **2-Sn**.

	**2-Ge**·2.5THF	**2-Sn**·2THF
Formula	$C_{81}H_{130}GeN_2NaO_{9.5}$	$C_{79}H_{126}N_2NaO_9Sn$
M	1379.44	1389.49
Cell setting	Orthorhombic	Monoclinic
Space group	*P*ccn	*P*n
a/Å	22.1979(9)	24.8337(10)
b/Å	25.5324(8)	13.8582(3)
c/Å	29.4423(12)	25.9967(9)
α/°	90	90
β/°	90	117.993(5)
γ/°	90	90
V/Å^3	16686.9(11)	7900.1(6)
Z	8	8
Unique/$I > 2\sigma I$	16330/10234	21614/17590
R_{int}	0.0438	0.0663
Parameters	1156	1639
R_1 (all data/$I > 2\sigma I$)	0.1746/0.1302	0.0912/0.0743
wR_2 (all data/$I > 2\sigma I$)	0.3830/0.3517	0.2190/0.1963
GooF	1.058	1.038
Residual max/min	0.712/–0.656	0.868/-1.244
T/K	150.0(2)	150.0(2)
CCDC Deposition No.	2141418	2141419

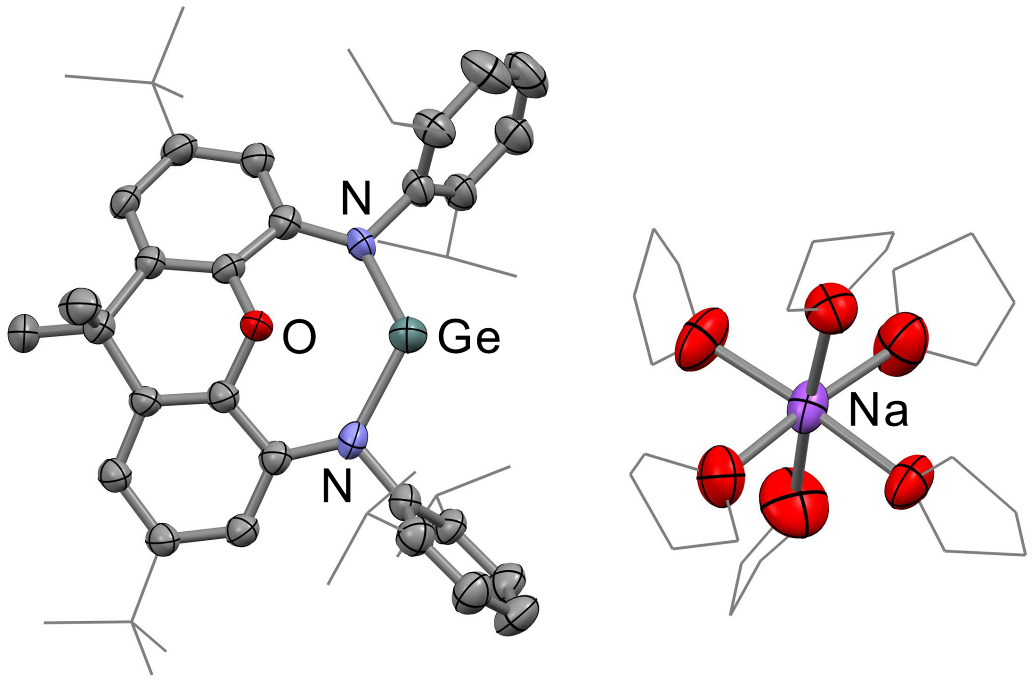

Figure S2.1. *Molecular structure of* ***2-Ge*** *as determined by X-ray crystallography. THF solvate molecules and hydrogen atoms have been omitted, and select groups shown in wireframe for clarity. Thermal ellipsoids set at the 50% probability level. Key bond lengths (Å) and angles (°): Ge-N 2.089(5), 2.072(6); Ge···O 2.641(4), Na-O 2.380(7), 2.396(8), 2.374(7), 2.347(11), 2.415(17), 2.358(9); N-Ge-N 117.2(2).*

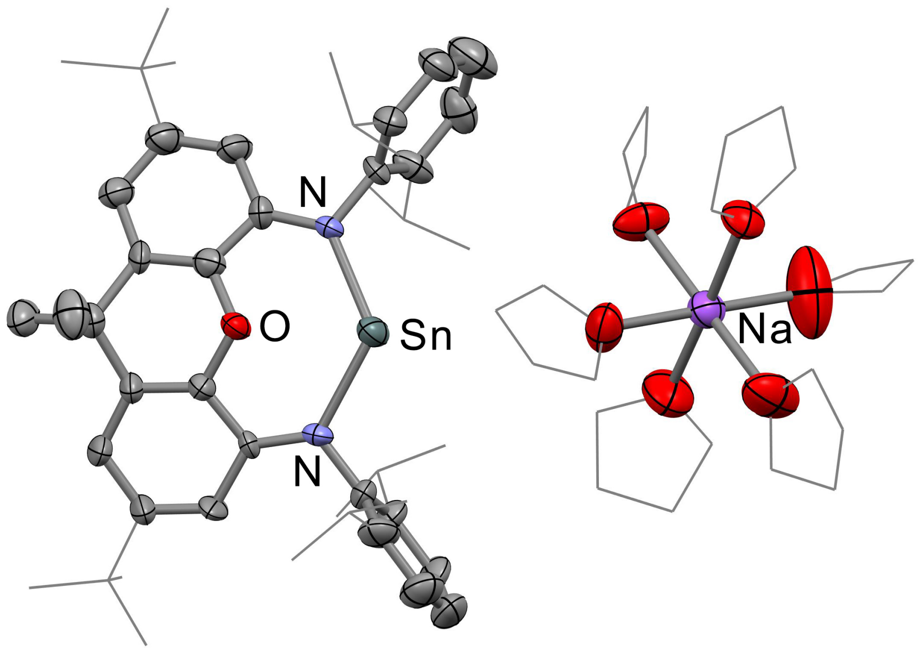

Figure S2.2. *Molecular structure of* ***2-Sn*** *as determined by X-ray crystallography. Only one of the two charged separated pairs in the ASU shown. THF solvate molecules and hydrogen atoms have been omitted, and select groups shown in wireframe for clarity. Thermal ellipsoids set at the 50% probability level. Key bond lengths (Å) and angles (°): Sn-N 2.260(11), 2.281(10); Ge···O 2.641(4), Na-O 2.345(18), 2.446(19), 2.34(2), 2.40(2), 2.41(2), 2.35(2); N-Sn-N 120.2(4).*

S3) EPR Materials and Methods

S3.1 EPR sample preparation.

The quartz EPR tube was first placed under vacuum, flushed three times with argon and cooled to −78 °C before a solutions of **2-Ge** or **2-Sn** was transferred into the tube. We note that best results were achieved by pre-cooling the transfer cannula by passing through just-above-freezing THF immediately before transfer. The transfer of **2-Pb** into an EPR tube proved challenging, with the procedure described above leading to the formation of a significant amount of metallic lead, which prevented the acquisition of any meaningful spectra. Improved results were achieved by cooling the transfer cannula with liquid dinitrogen immediately before it was used.

S3.2 EPR Measurements

Continuous Wave (CW) EPR measurements were performed on a Bruker E580 spectrometer equipped with an ER4122 SHQ resonator, an Oxford instruments E900 cryostat and ITC 4 temperature controller. The spectrum of the **2-Ge** complex was recorded at 80 K with a field modulation amplitude of 0.05 mT and a power of 0.047 mW. The spectrum of the **2-Sn** complex was recorded at 120 K with a field modulation amplitude of 0.05 mT and a power of 0.63 mW. The spectrum of the **2-Pb** complex was recorded at 10 K with a field modulation amplitude of 1.0 mT and a power of 0.047 mW.

Pulse EPR measurements were performed between 10-30 K using a Bruker dielectric resonator (MD5), Oxford instruments CF935 liquid helium cryostat and ITC-503 temperature controller. Electron spin echo-detected (ESE) field-swept spectra were measured using the pulse sequence: t_p–τ–$2t_p$–τ–*echo*. The length of $\pi/2$ microwave pulse was generally set to t_p = 8 ns. The interpulse distance was varied in the range τ = 120-500 ns. Three pulse ESEEM spectra were collected using the t_p–τ–t_p–t_1–t_p–τ–echo sequence with t_p 8 ns, τ = 196, 252, 418 ns and t_1 = 100-8000 ns in 8 ns steps. HYSCORE spectra were collected using the the t_p–τ–t_p–t_1–$2t_p$–t_2–t_p–τ–echo sequence with t_p 8 ns, τ = 196, 252, 418 ns and t_1, t_2 = 100-4408 ns (**2-Ge, 2-Sn**) and 100-1624 ns (**2-Pb**) in 8 ns (**2-Ge**), 12 ns (**2-Pb**) or 16 ns (**2-Sn**).

S3.3 Spin Hamiltonian Simulations

EPR spectra were simultaneously fit assuming a spin *S* = ½ ground state. The electron Zeeman, nuclear Zeeman and hyperfine terms were treated exactly. Spectral simulations were performed numerically using the EasySpin package[38,39] in MATLAB. The g tensor, metal hyperfine tensor were assumed to be collinear. An anisotropic linewidth was used for all simulations. Linewidth parameters (Sys.lw, Sys.HStrain) used are tabulated below. All other parameters can be found in Tables S5.1 and S5.3. For the simulation of three pulse ESEEM and HYSCORE data the two equivalent ^{14}N hyperfine tensors collinear with the g-tensor of the system were

sufficient to reproduce all spectral features. Inclusion of orientation selection did not significantly improve the fitting.

Table S3.3. EPR linewidth parameters

	Gaussian /mT	Lorentzian /mT	Anisotropic broadening / MHz
**2-Ge**	0	0.77	[14.7 62.3 0]
**2-Sn**	0	1.2	[0 56.5 86.6]
**2-Pb**	6.6	1.9	[294 240 0]

S4) Additional computational data

S4.1 Computational details

Geometry optimizations and calculations of spectroscopic parameters were performed using ORCA.[S9] The crystallographic coordinates were used as input for unconstrained geometry optimizations with the TPSS functional, while the hybrid TPSSh functional was used for calculation of spectroscopic properties owing to its well-documented performance for EPR parameters of open-shell systems. Optimizations were carried out both with and without the latest generation of dispersion corrections by Grimme and coworkers (D4).[S10] First-principles calculations of core properties such as the hyperfine coupling interaction necessitate the use of flexible all-electron basis sets to describe the distribution of charge and spin density close to the nucleus, while the nature of the target properties and the atomic weight of the elements involved require an adequate treatment of relativistic effects.[S11-S12] For these reasons we adopted the zero-order regular approximation (ZORA)[S13-S15] as a scalar relativistic Hamiltonian, in combination with all-electron ZORA-compatible basis sets. These were the purpose-made segmented all-electron relativistically contracted (SARC-ZORA) basis sets of valence TZVP quality for Sn and Pd,[S16-S17] and the ZORA-recontracted[S18] def2-TZVP basis sets[S19] for lighter atoms, including Ge, with the exception of C and H for which the ZORA-def2-ZVP basis set was used. Universal auxiliary basis sets (SARC/J) by Pantazis and coworkers were used for Coulomb fitting in the resolution of the identity approximation. Optimizations were performed with high integration accuracy ("DefGrid2"), which was further increased for the calculation of EPR parameters ("DefGrid3", combined with locally dense grids on the centers of interest). The hyperfine coupling calculations considered all contributions to the hyperfine coupling tensor, i.e. the Fermi contact term, the spin dipolar, and the spin-orbit coupling components. Picture-change effects were included in the calculation of EPR parameters. The mean-field approximation to the Breit–Pauli operator[S20-S22] was used as the effective spin–orbit coupling operator.

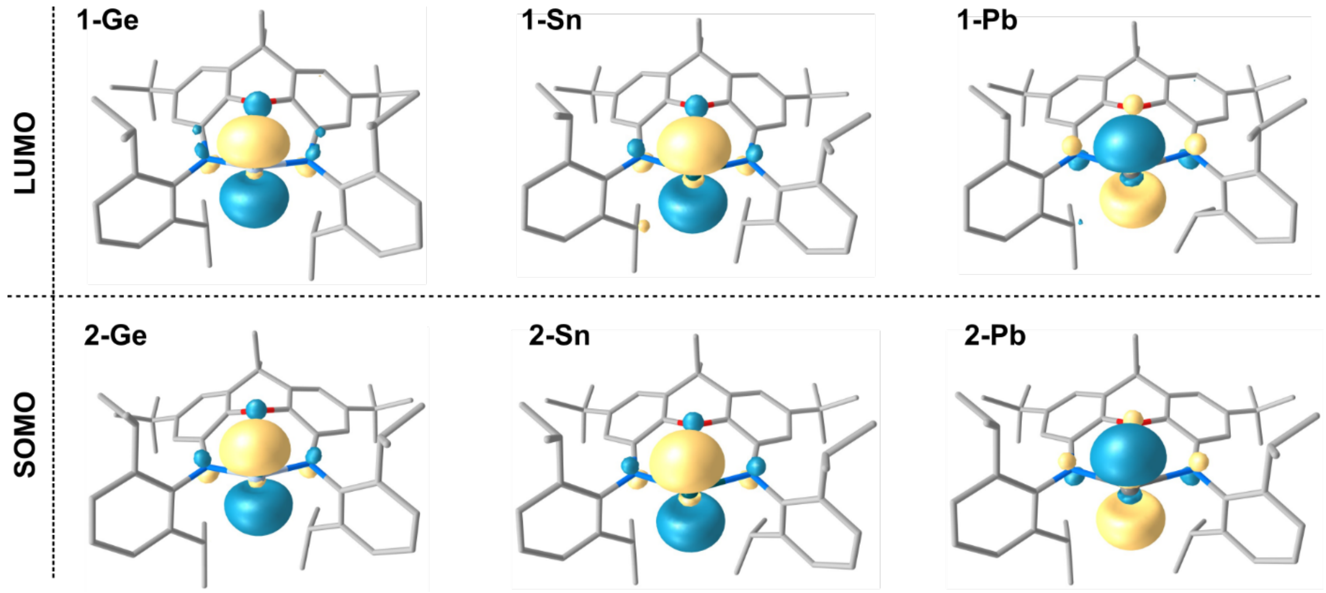

Figure S4.2. Frontier Molecular Orbitals of **1-Ge**, **1-Sn**, **1-Pb**, **2-Ge**, **2-Sn** and **2-Pb**.

Table S4.3. Comparison of crystallographic and DFT structural data (bond lengths Å, angles in degrees)

	**2-Ge**		**2-Sn**		**2-Pb**
	Exp.	DFT	Exp.	DFT	DFT
E–N(1)	2.090(5)	2.097	2.257(8)	2.301	2.404
E–N(2)	2.072(5)	2.085	2.280(10)	2.290	2.390
E···O	2.641(4)	2.667	2.610(10)	2.641	2.704
N(1)–E–N(2)	117.2(2)	115.0	120.2(4)	117.5	116.6

S5) Tabulated EPR and DFT data. Comparison to literature

Fitted spin Hamiltonian parameters for **2-Ge**, **2-Sn** and **2-Pb** are listed in Table S5.1 below. In all fittings, the g and metal hyperfine tensors were assumed to be collinear. For **2-Ge**, the metal hyperfine structure is poorly resolved. As such two fitting were attempted, one where the hyperfine tensor was allowed to vary all three principal components independently (rhombic symmetry) and one where two of the principals were constrained to be the same (axial symmetry). For **2-Sn** and **2-Pb** all spin Hamiltonian parameters are well defined, although the sign of the hyperfine components is ambiguous. In Table S5.1 they are chosen to match those calculated using DFT.

Fitted spin Hamiltonian parameters for each complex was compared against three DFT model types: I) a complete model inclusive of dispersion corrections; II) a model that excluded dispersion corrections; and III) a model inclusive of dispersion corrections, but which lacked the flanking N-coordinating Dipp groups. Type I models accurately reproduces all crystallographic constraints as shown in Table S4.3. For type II models, the ligand adopts a planar conformation unlike the hinged conformation seen for the two crystal structures. Type III models resemble type I models, with the exception that the molecule is now symmetric about a mirror plane that bisects the ligand containing the E···O interaction. In the two crystal structures, and for all type I models, the Dipp ligands adopt two slightly different orientations due to crystal packing, breaking the symmetry of the molecule. In frozen solution (EPR) measurements the symmetry of the molecule is expected to be retained.

All DFT model sets show good agreement with the experimental hyperfine tensors datasets. Calculated metal hyperfine tensor are essentially invariant for all three model sets demonstrating this parameter is insensitive to the precise conformation of the ligand (hinge angle, flanking N-coordinated substituents etc.) - or at least the degree of bending seen is insufficient to cause much effect. This is perhaps unsurprising. As described in the main text, the isotropic component of the hyperfine tensor is mainly derived from the Fermi contact term (A_{FC} spin density at the nucleus) and, as such, reports on the s orbital character of the SOMO. The A_{FC} for all DFT models was estimated to be very small, as compared to the maximum value (i.e. 100% *s* orbital character),[S23] consistent with the unpaired spin being localized in an un-hybridised metal p-orbital, which is orientated perpendicular to the plane of the ligand i.e. spin density is located above and below the plane of the ligand. This also likely explains the good agreement seen for between **2-Ge** and the earlier reported, planar Ge(I) radical from the group of Jones.[S24]

While the metal hyperfine tensor cannot discriminate between the three DFT model sets, the g tensor arguably can, although the effect is subtle. Here model set I and set III best reproduce the experimental values, with model set II systemically predicting a more rhombic g tensor for all complexes. As corrections to the g tensor for these complexes primarily stem from spin-orbit coupling (SOC) induced mixing within the p-orbital

manifold, this parameter is more sensitive to changes in the bonding of the complex that result from bending of the ligand backbone, and to a lesser extent loss of the flanking substituents.

Looking at each complex individually, we see that the DFT calculated parameters for **2-Ge** show the best agreement with experiment, matching the g tensor and anisotropic hyperfine term. All slight over-estimate the A_{iso} (30 MHz), which is approximately zero experimentally. We again emphasize though that this difference is negligible when considered as a percentage of the maximum possible hyperfine coupling value i.e. A_{max}(Ge) ≈ 2300 MHz, with $\Delta A = (A_{exp}-A_{max}) = 1.3\%$. Which is simply to say that any small change in the s-orbital character of the SOMO can have a dramatic effect on the calculated metal hyperfine couplings.

Similar good agreement is seen for **2-Sn** and **2-Pb**. Here the isotropic g-values are overestimated, particularly for **2-Pb**. Beyond this there are no clear trends: all models for **2-Sn** slightly over-estimate A_{iso} but under-estimate A_{aniso}, whereas for **2-Pb** the reverse is true, with A_{iso} underestimated, A_{aniso} overestimated. As with the **2-Ge** complex, the difference between the calculated and experimental values are very small when considered as a percentage of the maximum hyperfine coupling value and thus highly sensitive to any change in *s*-orbital character i.e. the maximum isotropic values for Sn and Pb are: A_{max}(Sn) ≈ 44000 MHz, A_{max}(Pb) ≈ 81500 MHz and thus the percentage difference is of the order of 0.5-2%.[S23]

No hyperfine structure could be assigned for NMR active nuclei of the ligand, consistent with little spin delocalization onto the ligand. They could however be observed using double resonance techniques such as Electron Spin Echo Envelope Modulation (ESEEM) and HYperfine Sublevel CORElation (HYSCORE). Only a weak, narrow signal was observed at the ^{1}H Larmor frequency [$\nu(^{1}H)$ = 14.9 MHz, 350 mT] in ESEEM traces for all three complexes (Figures S6.2 and S6.5). In the corresponding HYSCORE spectra (Figures S6.1, S6.4 and S6.7), a ridge (3 MHz peak-to-peak) was observed at the same frequency in the (+,+) quadrant, perpendicular to the diagonal, consistent with weakly (dipolar) coupled ^{1}H nuclei; a coupling of 3 MHz is equivalent to a metal-^{1}H inter-spin distance of ≈3 Å. This is approximately the distance between the metal center and the hydrogens of the pendant Dipp groups (see Figures S6.1-7).

All ESEEM and HYSCORE spectra were instead dominated by intense ^{14}N signals. Note that ESEEM relies on the pumping of spin forbidden transitions. These gain intensity when the cancellation condition is met i.e. when the hyperfine coupling (A) is twice the nuclear Lamor frequency (ν_I). Note too that as ^{14}N is a quadrupolar nucleus (I =1) it gives rise to multiple resonances.

The easiest feature to identify in these spectra is a doublet associated with the two double quantum transitions of the spin manifold (ν_α, ν_β). These appear at higher frequencies and are typically sharp and are described by the following analytical expressions:

$$\nu_\alpha = \pm 2\sqrt{\left(\nu_I + {}^{A}/_{2}\right)^2 + K^2(3+\eta^2)}$$

$$\nu_\beta = \pm 2\sqrt{\left(\nu_I - {}^{A}/_{2}\right)^2 + K^2(3+\eta^2)}$$

Where ν_I is the nuclear Larmor frequency [$\nu(^{14}N)$ = 1.077 MHz, 350 mT], A is the hyperfine coupling, K is the quadrupole coupling divided by 4 ($e^2qQ/4h$) and η the quadrupole asymmetry parameter.[S25-S26] Using these expressions rough estimates can be made of the hyperfine and quadrupole coupling constants which were used as a starting point for full simulations (see section S3) of the ESEEM and HYSCORE datasets.

Simulations of ESEEM and HYSCORE data for **2-Ge** are shown in Figures S6.2 and S6.3. A single ^{14}N coupling (i.e. a single nitrogen environment) rationalizes all spectral features. The fitted hyperfine and quadrupole coupling values are typical for ligands of first row transition metal complexes (Table S5.3).[S27] Interestingly, high frequency correlations marked with an asterisk in Figure S6.3 are indicative of two equivalent nitrogen environments i.e. the molecule does recover its mirror plane in solution.

Corresponding simulations of ESEEM and HYSCORE data for **2-Sn** are shown in Figures S6.5 and S6.6. As with **2-Ge**, a single ^{14}N coupling/environment rationalizes all spectral features. The hyperfine coupling for **2-Sn** is slightly smaller than that of **2-Ge** (3.4 vs. 2.4 MHz), but the quadrupole coupling is the same. The fitted values show good agreement with those generated from DFT models described above, although the isotropic hyperfine coupling is systematically underestimated by ≈ 2 MHz

A full analysis of ^{14}N ESEEM and HYSCORE data of **2-Pb** was not achieved in this study. Preliminary analysis (Figure S6.7) though indicates that the ^{14}N hyperfine coupling for this complex is approximately two-fold larger than that seen for both **2-Ge** and **2-Sn**, but still below 10 MHz. As before, the quadrupole coupling is of the order of 3.5 MHz.

Table S5.1. Fitted spin Hamiltonian parameters for **2-Ge**, **2-Sn** and **2-Pb**. Comparison to calculated EPR parameters. All hyperfine values are given in MHz.

		g_{xx}	g_{yy}	g_{zz}	g_{iso} [g)]	g_{aniso} [h)]	g(η) [i)]	A_{xx}	A_{yy}	A_{zz}	A_{iso} [g)]	A_{aniso} [h)]	A(η) [i)]
**2-Ge**	Exp. [a)]	1.976	1.976	1.999	1.984	+0.008	0.05	0.7 [c)]	58.7	-73.0	-4.6	-34.2	0.85
	Exp. [b)]	1.976	1.976	1.999	1.984	+0.008	0.05	38.5	38.5	-75.5	0.5	-38.0	0.00
	DFT [d)]	1.977	1.979	2.002	1.986	+0.008	0.16	60.5	58.8	-48.1	23.7	-35.9	0.02
	DFT [e)]	1.955	1.982	2.002	1.980	-0.012	0.81	70.5	69.2	-36.3	34.5	-35.4	0.02
	DFT [f)]	1.964	1.977	2.003	1.981	+0.011	0.60	58.7	56.9	-51.5	21.4	-36.4	0.02
**2-Sn**	Exp.	1.879	1.906	1.982	1.922	+0.030	0.46	1411	1426	-1287	517	-902	0.01
	DFT [d)]	1.905	1.921	2.002	1.943	+0.030	0.28	1554	1542	-498	866	-682	0.01
	DFT [e)]	1.883	1.931	2.002	1.939	+0.032	0.76	1621	1547	-456	904	-680	0.05
	DFT [f)]	1.886	1.917	2.003	1.935	+0.034	0.46	1579	1536	-518	866	-692	0.03
**2-Pb**	Exp.	1.335	1.410	1.693	1.479	+0.107	0.35	-5598	-6008	-3180	-4929	+874	0.23
	DFT [d)]	1.592	1.728	1.998	1.773	+0.113	0.60	-4211	-4149	-823	-3131	+1540	0.12
	DFT [e)]	1.548	1.751	1.998	1.767	+0.116	0.87	-4571	-4117	-868	-3185	+1159	0.20
	DFT [f)]	1.540	1.726	2.001	1.756	+0.123	0.76	-4506	-4148	-772	-3142	+1185	0.15

[a)] best fit assuming $A_{xx} \neq A_{yy} \neq A_{zz}$ i.e. hyperfine tensor is rhombic; [b)] best fit assuming $A_{xx}=A_{yy} \neq A_{zz}$ i.e. hyperfine tensor is axial; [c)] Ill defined – within the experimental linewidth; [d)] DFT model including dispersion corrections i.e. correctly reproduces the hinged structure; [e)] DFT model excluding dispersion corrections i.e. the ligand is planar; [f)] Truncated DFT model including dispersion corrections i.e. symmetric about the C_s mirror plane; [g)] $g_{iso} = (g_{xx}+g_{yy}+g_{zz})/3$; [h)] $g_{aniso} = g_c/2$; where $[g_a\ g_b\ g_c] = ([g_1\ g_2\ g_3] - g_{iso})$. The principal tensor values (g_{xx}, g_{yy}, g_{zz}) are reordered such that $|g_1| \leq |g_2| \leq |g_3|$; [i)] $g(\eta) = (g_a - g_b)/g_c$.

Table S5.2. Decomposition of calculated metal centered hyperfine tensors for 2-Ge, 2-Sn and 2-Pb for DFT model set including dispersion corrections.

	A_{FC}	A_{SD}			A_{SO}			A_{Tot}		
		x	y	z	x	y	z	x	y	z
**2-Ge**	19.2	34.8	32.7	–67.5	6.5	6.9	0.1	60.5	58.8	–48.1
**2-Sn**	600	540	562	–1102	413	380	4	1553	1542	–498
**2-Pb**	-1913	–562	–566	1128	–1945	–1669	–38	–4420	–4148	–823

Table S5.3. Calculated ^{14}N hyperfine values. All hyperfine values are given in MHz.

	**2-Ge**		**2-Sn**	
	DFT	Exp.	DFT	Exp.
A_{xx}	-1.1	-4.4	-1.4	-3.8
A_{yy}	-2.2	-3.9	-1.5	-2.7
A_{zz}	-2.3	-1.9	2.3	-0.7
A_{iso}[a)]	-1.9	-3.4	-0.2	-2.4
A_{aniso}[b)]	+0.38	+0.75	+1.25	+0.85
A(η)[c)]	0.16	0.36	0.04	0.67
e^2qQ/h	-3.0	-3.5	3.0	3.4
$e^2qQ/h(\eta)$	0.95	0.84	0.75	0.81

[a)] $A_{iso} = (A_{xx}+A_{yy}+A_{zz})/3$; [b)] $A_{aniso} = A_c/2$; where $[A_a\ A_b\ A_c] = ([A_1\ A_2\ A_3] – A_{iso})$. The principal tensor values (A_{xx}, A_{yy}, A_{zz}) are reordered such that $|A_1| \le |A_2| \le |A_3|$; [c)] $A(\eta) = (A_a-A_b)/A_c$

Table S5.4. Ge(I) and Ge(III) EPR literature data[S24, S28-S30]

Compound	$[g_x\ g_y\ g_z]$	g_{iso}[a]	g_{aniso}[b]	η(g)[c]	$[A_x\ A_y\ A_z]$	A_{iso}[a]	A_{aniso}[b]	η(A)[c]
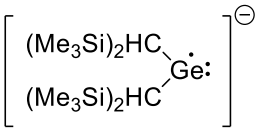	Not reported	2.0125	N/A	N/A	Not reported	35	N/A	N/A
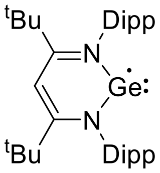	1.968<br>1.997<br>2.001	1.988	-0.010	0.198	82.5<br>37.5<br>42.0	54.0	+14.3	0.158
$[(Me_3Si)_2CH]_3Ge\cdot$	Not reported	2.0078	N/A	N/A	Not reported	258	N/A	N/A
$[(Me_3Si)_3N]_3Ge\cdot$	Not reported	1.9991	N/A	N/A	Not reported	479	N/A	N/A
$(^tBu_2MeSi)_3Ge\cdot$	Not reported	2.0229	N/A	N/A	Not reported	56	N/A	N/A

Table S5.5. Sn(I) and Sn(III) EPR literature data [S28-S29, S31-S35]

Compound	$[g_x\ g_y\ g_z]$	g_{iso}[a]	g_{aniso}[b]	η(g)[c]	$[A_x\ A_y\ A_z]$	A_{iso}[a]	A_{aniso}[b]	η(A)[c]
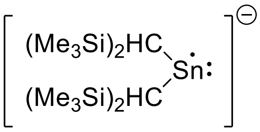	Not reported	2.0177	N/A	N/A	Not reported	325	N/A	N/A
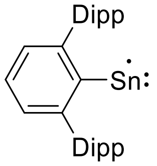	2.033<br>1.983<br>1.947	1.988	+0.023	0.794	Not reported	1603	N/A	N/A
$[(Me_3Si)_2CH]_3Sn\cdot$	Not reported	2.0094	N/A	N/A	Not reported	4868	N/A	N/A
$[(Me_3Si)_3N]_3Sn\cdot$	Not reported	1.9912	N/A	N/A	Not reported	9251	N/A	N/A
$(^tBu_2MeSi)_3Sn\cdot$	Not reported	2.0482	N/A	N/A	Not reported	922	N/A	N/A
$[(Me_3Si)_2EtSi]_3Sn\cdot$	2.080<br>2.079<br>1.985	2.048	-0.031	0.016	558<br>631<br>2587	1259	+664	0.055
$[(Me_3Si)_3Si]_3Sn\cdot$	2.074<br>2.074<br>1.991	2.046	-0.028	0	1041<br>1041<br>3087	1723	+682	0
$[(Me_3Si)_3Ge]_3Sn\cdot$	2.080<br>2.080<br>1.994	2.051	-0.029	0	1317<br>1205<br>3139	1887	+626	0.090

Table S5.6. Pb(III) EPR Literature data [S34-S36]

Compound	[g_x g_y g_z]	g_{iso}[a]	g_{aniso}[b]	η(g)[c]	[A_x A_y A_z]	A_{iso}[a]	A_{aniso}[b]	η(A)[c]
$[(Me_3Si)_2EtSi]_3Pb\cdot$	2.246<br>2.245<br>1.888	2.126	-0.119	0.004	390<br>429<br>4041	1620	+1211	0.016
$[(Me_3Si)_3Si]_3Pb\cdot$	2.237<br>2.213<br>1.893	2.114	-0.111	0.108	1177<br>1233<br>4904	2438	+1233	0.023
$[(Me_3Si)_3Si]_2[(Me_3Si)_3Ge]Pb\cdot$	2.294<br>2.011<br>1.699	2.001	-0.152	0.936	617<br>2410<br>2326	1784	-584	0.072
$[(Me_3Si)_3Si][(Me_3Si)_3Ge]_2Pb\cdot$	2.309<br>2.226<br>1.687	2.074	-0.194	0.215	280<br>1738<br>2046	1354	-537	0.287
$[(Me_3Si)_3Ge]_3Pb\cdot$	2.293<br>2.292<br>1.667	2.084	-0.209	0.002	953<br>981<br>1962	1299	+332	0.042
$Me_3Pb\cdot$	2.106<br>2.097<br>1.914	2.039	-0.603	0.072	3223<br>3223<br>9080	5175	+1952	0

a) $g_{iso} = (g_{xx}+g_{yy}+g_{zz})$

b) $g_{aniso} = g_c/2$; where $[g_a\ g_b\ g_c] = ([g_1\ g_2\ g_3] - g_{iso})$. The principal tensor values (g_{xx}, g_{yy}, g_{zz}) are reordered such that $|g_1| \leq |g_2| \leq |g_3|$

c) $g(\eta) = (g_a - g_b)/g_c$

S6) Additional Pulse EPR data

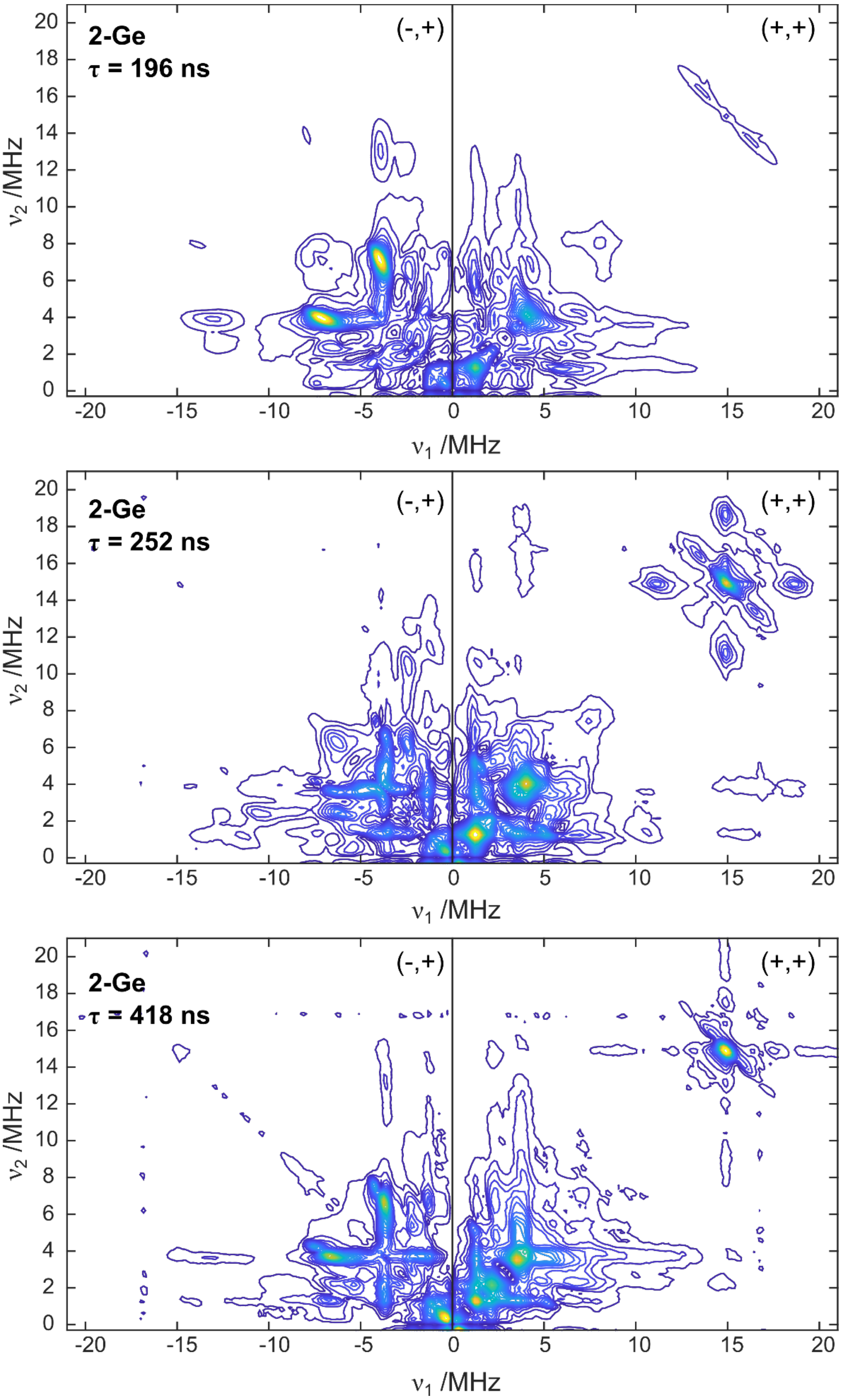

Figure S6.1. X-band HYSCORE spectra of **2-Ge** recoded in the center of its EPR spectrum (350 mT). All EPR parameters are listed in S3.2. The ridge centered at ≈15 MHz in the (+,+) quadrant is consistent with weakly (dipolar) coupled ^{1}H nuclei; a coupling of 3 MHz is equivalent to a metal-^{1}H inter-spin distance of ≈3 Å. This is approximately the distance between the metal center and the hydrogens of the pendant Dipp groups. The two intense cross-peaks in the (-, +) quadrant centered at (-3.9, -7.1) and (-7.1, -3.9) MHz are consistent with the double quantum transitions of a ^{14}N nucleus and consistent with a hyperfine coupling of A ≈ 4.0 MHz, see section S5.

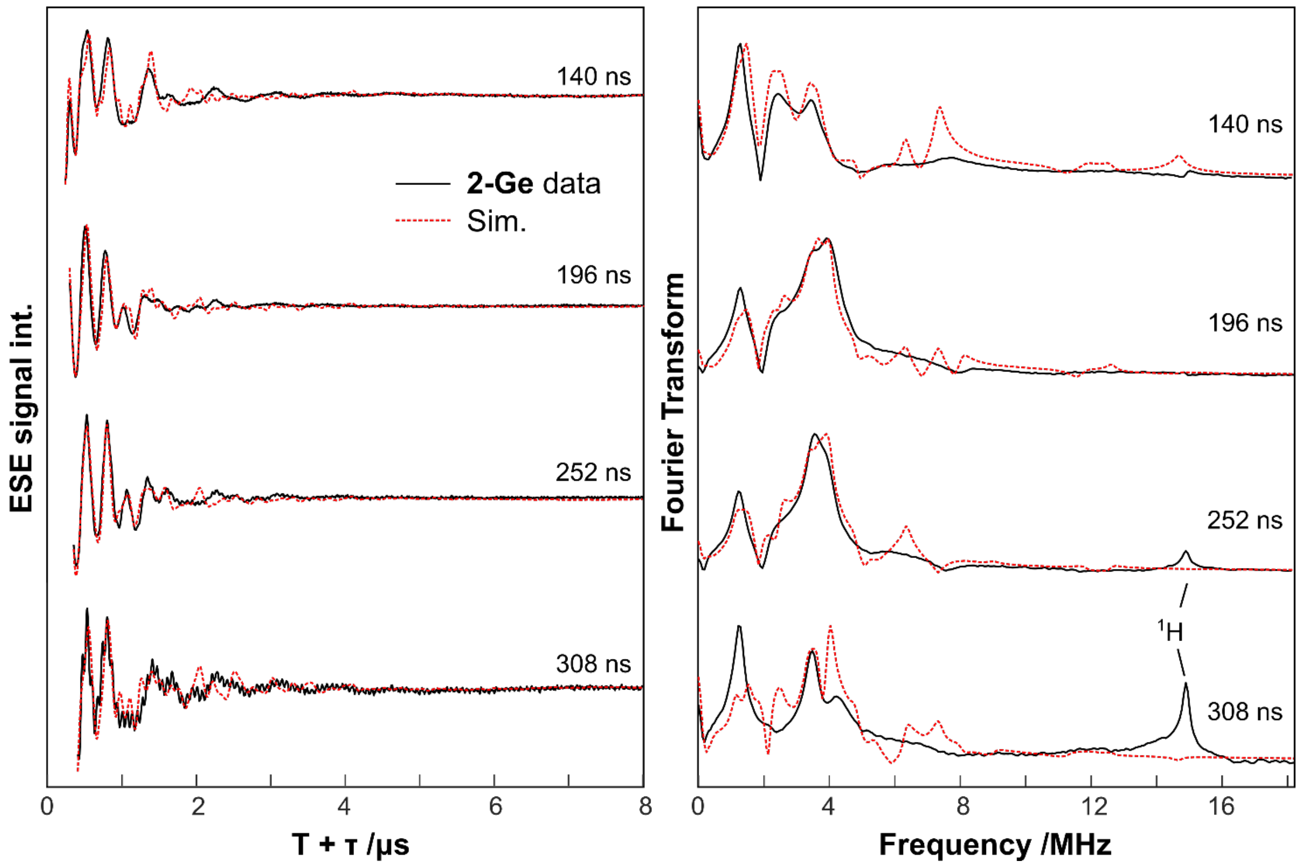

Figure S6.2. X-band three pulse ESEEM spectra of **2-Ge** recoded in the center of its EPR spectrum (350 mT). All EPR parameters are listed in S3.2. The black line shows the data, the red line a simulation using the spin Hamiltonian formalism. The simulation is described in S3.3 and all fitted parameters listed in Table S5.3.

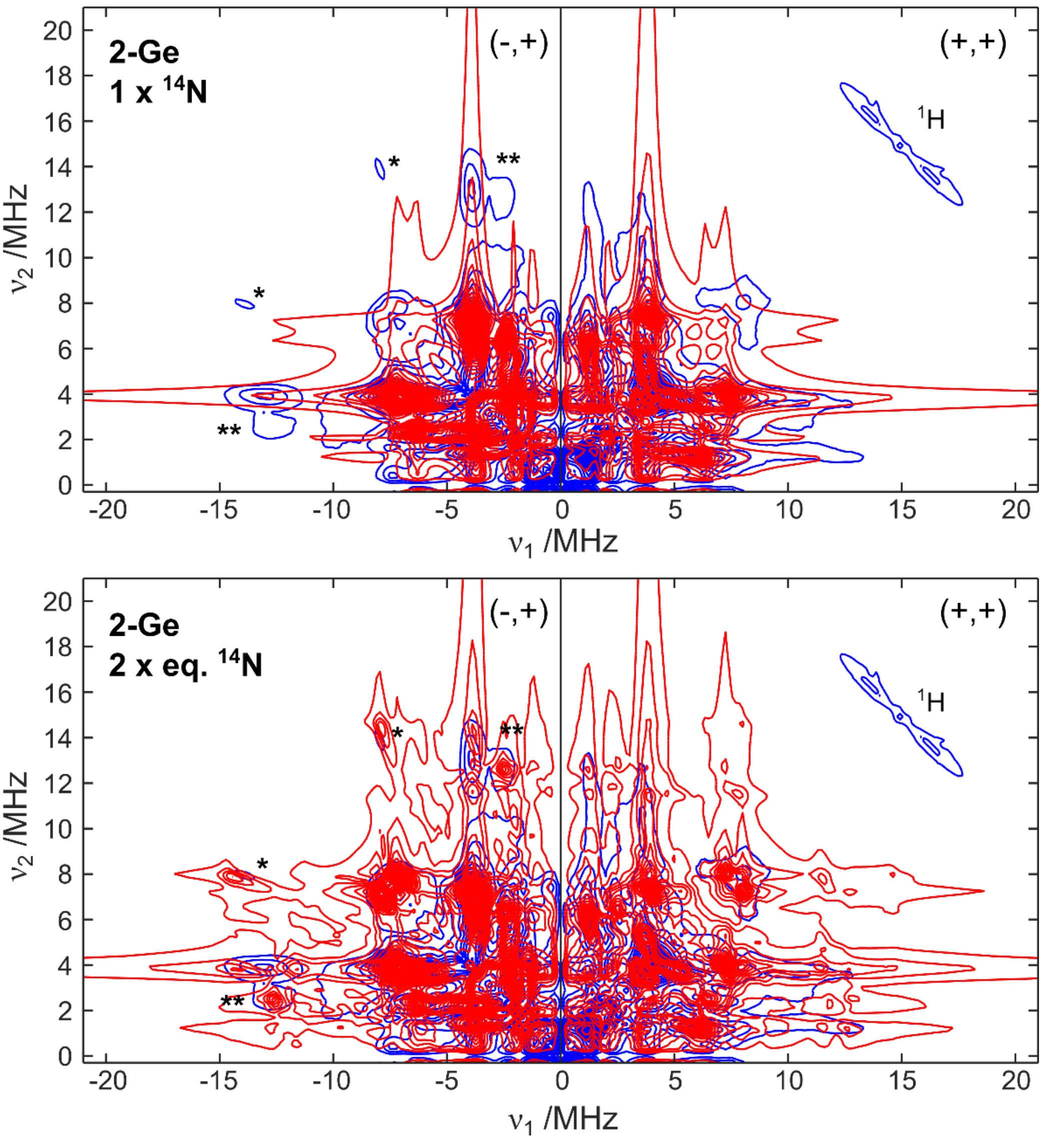

Figure S6.3. X-band HYSCORE spectrum (τ = 196 ns) of **2-Ge** superimposed with a spin Hamiltonian simulation using the same parameters as in Figure S6.2. The **top** simulation includes only one ^{14}N nucleus whereas the **bottom** simulation includes two equivalent ^{14}N nuclei. Note that while both simulations reproduce the intense cross peaks in the center of the spectrum, only the bottom simulation reproduces weaker high frequency correlations marked with an asterisk e.g. the frequency correlations at (-13, -3.9) and (-3.9, -13) MHz etc. that appear at twice the frequency of the prominent double quantum ^{14}N peaks are indicative of two equivalent nitrogen environments. The simulation is described in S3.3 and all fitted parameters listed in Table S5.3.

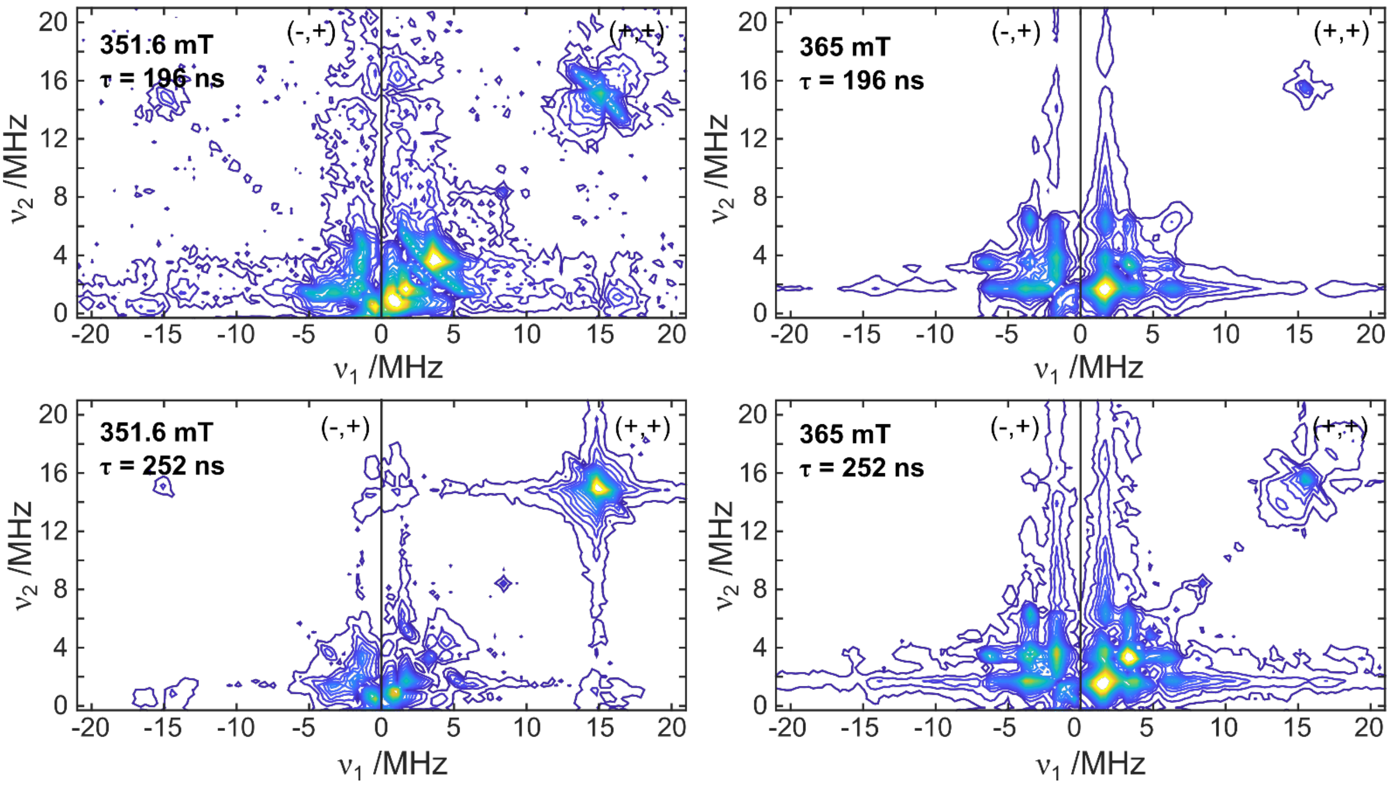

Figure S6.4. X-band HYSCORE spectra of **2-Sn** recoded on the low field edge (351.6 mT) and in the center (365 mT) of its EPR spectrum. All EPR parameters are listed in S3.2. As above, the ridge centered at ≈15 MHz in the (+,+) quadrant is consistent with weakly (dipolar) coupled ^{1}H nuclei; a coupling of <3 MHz is equivalent to a metal-^{1}H inter-spin distance of <3 Å. This is approximately the distance between the metal center and the hydrogens of the pendant Dipp groups. The two intense cross-peaks in the (-, +) quadrant cenetred at (-3.4, -6.5) and (-6.5, -3.4) MHz are consistent with the double quantum transitions of a ^{14}N nucleus with a hyperfine coupling A ≈ 3 MHz.

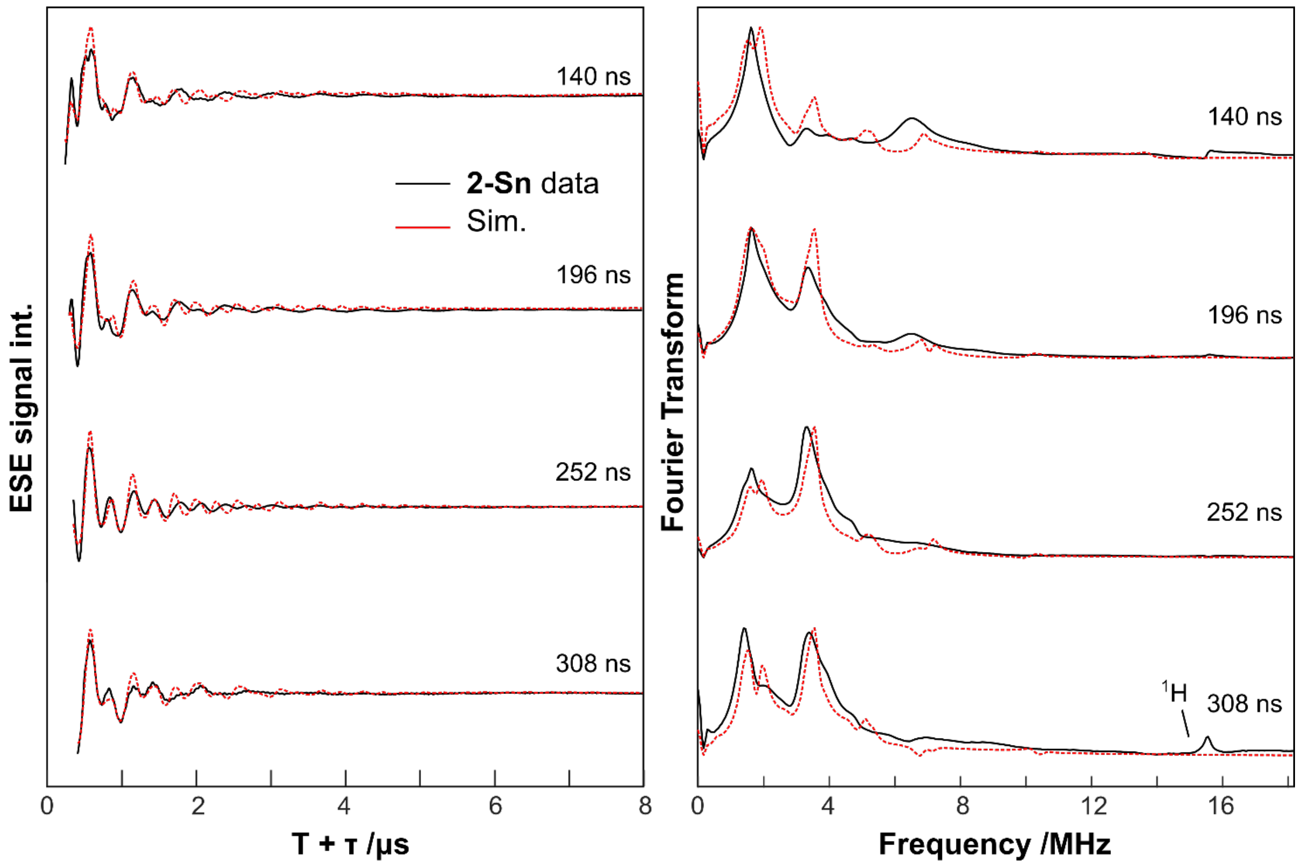

Figure S6.5. X-band three pulse ESEEM spectra of **2-Sn** recoded in the center of its EPR spectrum (365 mT). All EPR parameters are listed in S3.2. The black line shows the data, the red line a simulation using the spin Hamiltonian formalism. The simulation is described in S3.3 and all fitted parameters listed in Table S5.3.

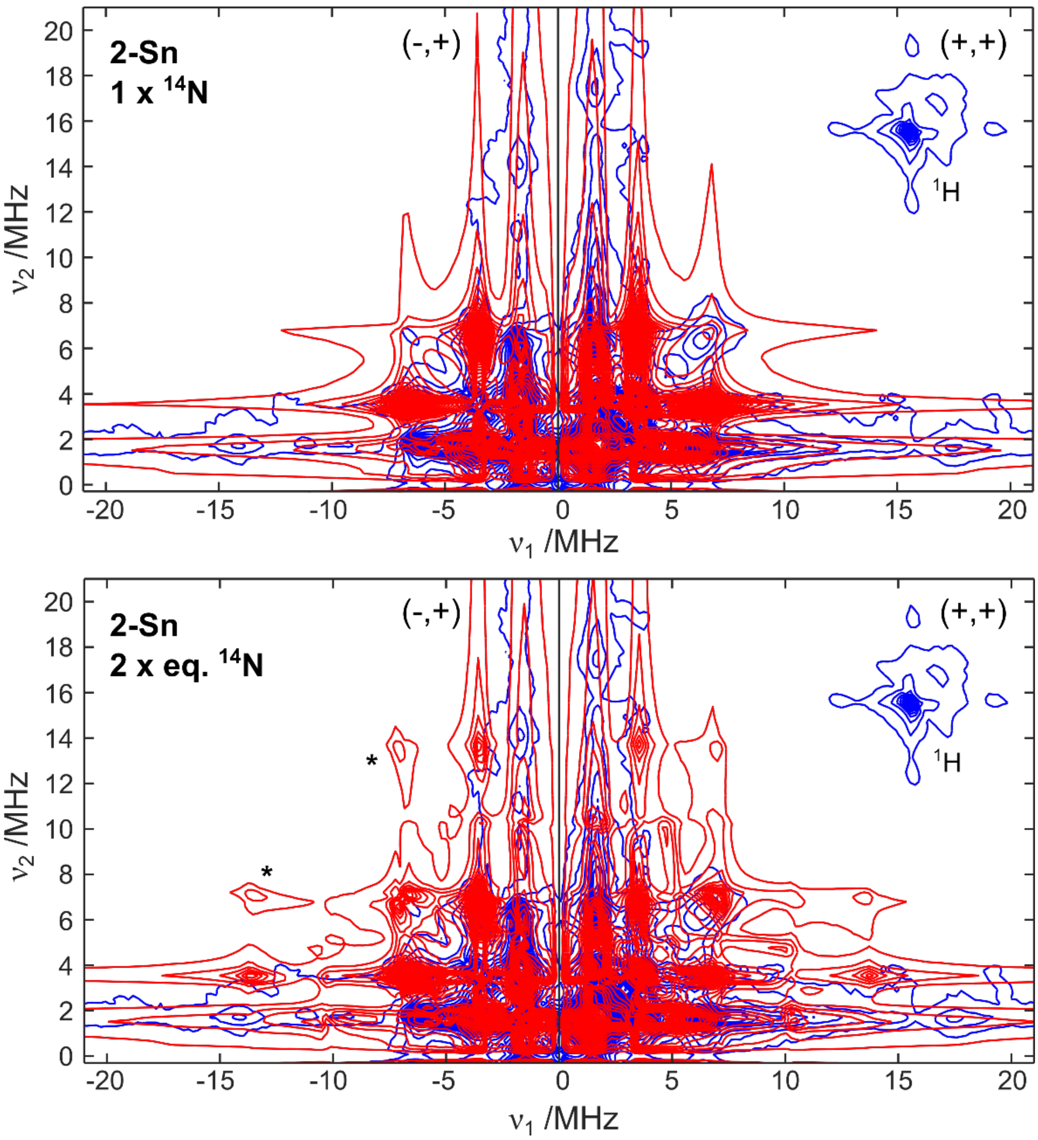

Figure S6.6. X-band HYSCORE spectrum (τ = 196 ns) of **2-Sn** superimposed with a spin Hamiltonian simulation using the same parameters as in Figure S6.5. The **top** simulation includes only one ^{14}N nucleus whereas the **bottom** simulation includes two equivalent ^{14}N nuclei. Owing to poorer data quality, higher frequency correlations are not clearly resolved and as such both fitting equally reproduce the data. We note however that we see no evidence for two nitrogen environments. The simulation is described in S3.3 and all fitted parameters listed in Table S5.3.

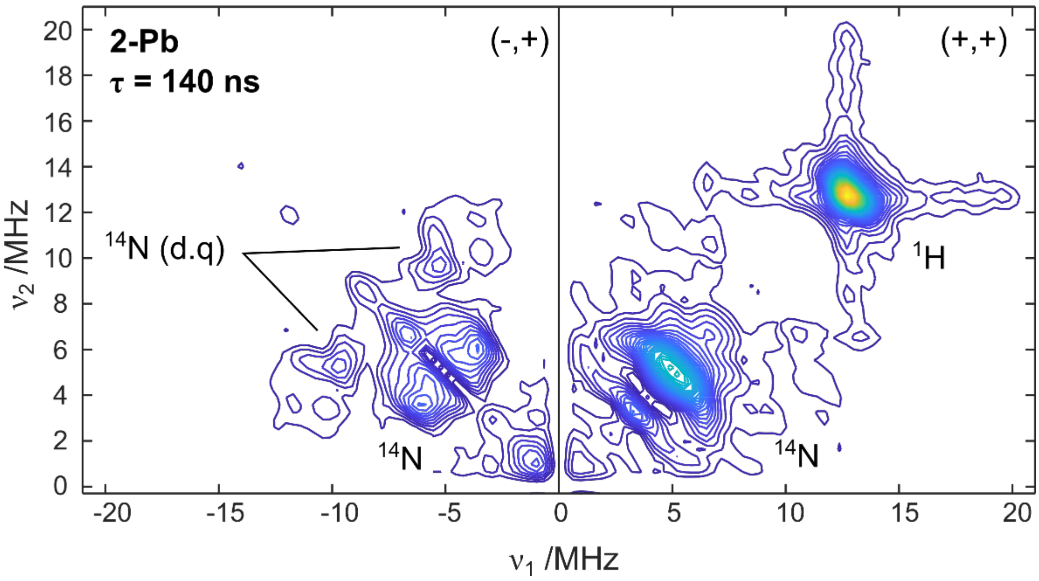

Figure S6.7. X-band HYSCORE spectrum of **2-Pb** recoded on the low field edge (298 mT) of its EPR spectrum. All EPR parameters are listed in S3.2. As above, the cross-peak centered at ≈15 MHz in the (+,+) quadrant is consistent with weakly (dipolar) coupled ^{1}H nuclei. Cross-peaks in the (-, +) quadrant cenetred at (-9.8, -5.2) and (-5.2, -9.8) MHz are consistent with the double quantum transitions of a ^{14}N nucleus with a hyperfine coupling of A ≈ 8 MHz.

S7) Cartesian coordinates of optimized structures for 2-Ge, 2-Sn and 2-Pb

2-Ge:

Ge	-0.11181607521463	-0.01349200026912	-0.02749216765031
O	-0.03881700625992	-0.00006046325963	2.63815955602512
N	0.60533179318470	1.77405776096210	0.80239594785905
N	0.66162321320471	-1.75171116440145	0.82517117992659
C	0.57620823088010	1.14396805230727	3.10956272748470
C	0.84555133537564	2.10805488762758	2.11454831802963
C	0.54337228042868	-1.15368955930607	3.12878442215932
C	2.10621965277380	-2.79816169769380	-0.87017640517964
C	0.91608916630386	-1.24072170525108	4.46176115967870
C	0.84367934025909	-2.11174454538913	2.13621538095322
C	0.65729851445608	0.00917862550737	5.32495633084199
C	1.49275484366932	3.05780470157977	-1.11138125091020
C	0.87810398157526	-2.75362013412195	-0.15429138151995
C	1.71745131411423	-3.50152682934859	3.98096071632425
C	1.55878904711020	2.44272943441373	4.86190028887540
H	1.86336063630097	2.55454731470305	5.90024026106967
C	0.95499130813598	1.23685099151469	4.44141662440519
C	1.76360716617672	3.49182303657442	3.94274917536852
C	0.49554710583253	2.84623426403639	-0.11831473472020
C	1.40779707883633	3.32012621515383	2.58847913426779
H	1.60737642470600	4.11133721076546	1.86419225273446
C	2.29899449518178	-3.81823345621861	-1.81599208995509
H	3.23818297662534	-3.86742459126795	-2.37014068387645
C	1.50668681506417	-2.44943445443963	4.89490568568887
H	1.79997003267059	-2.55839223474839	5.93670268229062
C	2.36171226963173	-4.83486326433312	4.41030572136255
C	3.19077114026750	-1.77242983318887	-0.56855264503634
H	2.66177720213517	-0.85567976970527	-0.26322015918931

C	1.38854221836043	-3.32904054606370	2.61953798947870
H	1.61456596795972	-4.11498176334692	1.89736422886877
C	-0.64205903093835	3.69991612378462	-0.05247387453326
C	-0.84563362171415	0.03910659094767	5.72423601649676
H	-1.48248482110632	0.04172183614019	4.83001234267904
H	-1.06490685902255	0.94435140176026	6.31062004727518
H	-1.09641826774316	-0.84652895351531	6.32772380648099
C	2.70327769600277	2.13400281261932	-1.17749803204796
H	2.31087596400315	1.11426774819795	-1.02075037599789
C	0.09654650741703	-4.71738925669719	-1.36247939473520
H	-0.68091935575507	-5.45948252039737	-1.56358904414340
C	1.34592038380029	4.14062330233379	-1.99478266066038
H	2.10407548177688	4.31761842586699	-2.75891790449176
C	-0.14101226510589	-3.70943933033789	-0.41125088423692
C	4.05924687967314	-1.43427514560826	-1.78981421734021
H	4.66718119446973	-2.29415197610553	-2.11358827182676
H	4.75533604602436	-0.62083704211638	-1.53878336899345
H	3.44170044845738	-1.10933350002059	-2.63903014653803
C	1.30759508713618	-4.77982940739454	-2.05914481659695
H	1.47868881601268	-5.56868777224858	-2.79511363122093
C	4.07069762576671	-2.21150456215182	0.62162874122074
H	3.46477723627759	-2.38895455656719	1.51847850301879
H	4.81084931273763	-1.43071868637704	0.85652658210316
H	4.61446336019628	-3.14025032599156	0.38405897312157
C	3.41521526719748	2.16437644436764	-2.53872401647768
H	2.71672747409888	1.94783293871833	-3.36023044140806
H	4.21447130956160	1.41160596338852	-2.56395411275938
H	3.88129071542746	3.14372432712008	-2.73326467383081
C	-0.74004036038113	4.77758993268302	-0.94860965711342
H	-1.61081331464035	5.43720141544012	-0.90022001362687
C	0.24715413338185	5.00628853811946	-1.91243210812185

H	0.15755411482864	5.84615852452741	-2.60507885104796
C	2.42279720842873	4.82192023941314	4.35916089062805
C	1.51623389306928	0.00615875102645	6.59971989689093
H	1.28259943229634	-0.87115353431338	7.21964809010186
H	1.31048716322764	0.90093066830395	7.20437347687294
H	2.58776171001549	-0.01302942205273	6.35739278994939
C	-1.47650195462485	-3.63881415341188	0.31853799115073
H	-1.42999905241407	-2.76959079445410	0.98874292237143
C	2.70962040783138	4.88598647933492	5.87280324997235
H	1.78675284793007	4.77382871911890	6.46073231829452
H	3.15972712172973	5.85841524291567	6.12620805935126
H	3.41166690791441	4.09831447262191	6.18307238892680
C	3.72129365347215	-5.00029842714416	3.68566085422647
H	3.59343385208992	-5.00455566261000	2.59461749486814
H	4.20616735383488	-5.94557264170612	3.98003334138728
H	4.39487733210351	-4.16907844645306	3.93974126949364
C	2.61557518509312	-4.90099276164554	5.92980796001199
H	3.31467457618799	-4.11692557357638	6.25540715878898
H	3.05590599217189	-5.87561825450133	6.19182823561715
H	1.68094080207514	-4.78469936433732	6.49797168467079
C	-1.76684761812724	3.43675491056254	0.94227251127752
H	-1.47689280834021	2.56553840382801	1.54308750535771
C	3.70005368368918	2.42322904607904	-0.03569747416929
H	4.11713264084431	3.43877979241205	-0.13053341011943
H	4.53517082778324	1.70596880691790	-0.06686997917758
H	3.21606169311198	2.33953925722846	0.94456406423157
C	-3.06958155982725	3.06200484415519	0.20597232429986
H	-3.41565725499763	3.88190496316206	-0.44336645547576
H	-3.86937778078870	2.83818884996643	0.92926900757937
H	-2.91067996842834	2.17042405157095	-0.41646100399117
C	1.49143592923983	6.00344134216550	3.99508554189909

H	1.28074626594070	6.02960459821622	2.91745180776782
H	1.95583355520252	6.96282137490204	4.27612652743603
H	0.53082240747429	5.91571237215505	4.52351733473396
C	1.43295787548720	-6.01160565809126	4.02498214977777
H	0.45788705667466	-5.91486769462828	4.52451412334087
H	1.88268856123097	-6.97295344616686	4.32270202863369
H	1.25412554562750	-6.04043612245897	2.94169922584957
C	-2.63521535063026	-3.39384288606883	-0.66921252382748
H	-2.46884526805188	-2.46528124531736	-1.23260525831192
H	-3.58993921858330	-3.30116044929818	-0.12828863537175
H	-2.73162818495396	-4.22162442284238	-1.38983350426463
C	3.76700822730212	4.97943168208566	3.60491769071653
H	4.44314054758538	4.14770229670227	3.85042861457878
H	4.26116420752698	5.92508921399985	3.88227220085242
H	3.61539246154930	4.97612433725030	2.51686783827230
C	-1.72412469782621	-4.89228808644214	1.18124273445716
H	-1.77971776914534	-5.80158963855814	0.56123775691953
H	-2.67371132293952	-4.80085459117273	1.73111171656863
H	-0.91723659836571	-5.02332815755899	1.91466306492254
C	-1.97834401737269	4.62265809356379	1.90390644180854
H	-1.05331404202206	4.84486644992203	2.45201819568506
H	-2.76289513029214	4.38559459340655	2.63903654956862
H	-2.28750793559758	5.53098519954475	1.36205203629619

2-Sn:

Sn	-0.12395710114467	-0.00961134872986	-0.13366142676621
O	0.29788784433155	-0.00025733043564	2.47334281479726
N	0.61650966093368	1.96744790242041	0.78202987365040
N	0.64517331955365	-1.95765655571532	0.79189453878644
C	0.76908657693111	1.17669778450163	3.02695173416463
C	0.89070386069251	2.23609211262727	2.09324553775839

C	0.78821895051035	-1.16721583530829	3.03214482946484
C	2.05217854074965	-3.05932400334125	-0.89004319114679
C	1.14547961692761	-1.23604839547084	4.37047903306003
C	0.94404105718080	-2.22070009491583	2.09707386772760
C	0.89953476832049	0.00846246761257	5.23814449059587
C	1.59729373125186	3.26484489581673	-1.05939627379497
C	0.83626437539649	-2.98869083633775	-0.15532030455898
C	1.78841088732778	-3.57005917280121	3.98522334961212
C	1.60048675821980	2.49727279501556	4.84440445210614
H	1.88379365486341	2.58869272709628	5.89018912510841
C	1.12265379773771	1.25484249573062	4.36645027889590
C	1.70227077690562	3.60510323951111	3.98334174767579
C	0.54700591474443	3.05047187716742	-0.12219650647066
C	1.33978319186962	3.47036100455827	2.62727101655971
H	1.44197050766885	4.31527199072903	1.94437301056647
C	2.20976440820689	-4.07981000176620	-1.84271510954338
H	3.14220747401940	-4.14691674777367	-2.40793902779819
C	1.65355208952585	-2.46697742732070	4.84820623728640
H	1.93397537071202	-2.55326332206674	5.89522376474301
C	2.32801956292628	-4.92758095811550	4.47616960898954
C	3.17721582895884	-2.07368311636745	-0.60334079982435
H	2.71979763724991	-1.22798454833344	-0.06828522312800
C	1.43238725681604	-3.44153669201139	2.62660350923501
H	1.55891433134381	-4.28138888180321	1.94154385569165
C	-0.59894837937655	3.89358947779069	-0.12586966203924
C	-0.58874549059784	-0.00378386747944	5.69396247521105
H	-1.25675227692001	-0.01089859102520	4.82239678980750
H	-0.81063459686674	0.89156401082425	6.29446959207962
H	-0.79498125788507	-0.90115755428471	6.29702830029875
C	2.82218530208768	2.35826768441082	-1.03593133615453
H	2.45320556518531	1.35015454106511	-0.78190495484865

C	-0.00402523530442	-4.93175507368896	-1.35937892538117
H	-0.79921630675315	-5.65732980759533	-1.55121251357435
C	1.49089238990627	4.33752020032937	-1.96073768816941
H	2.28968166784891	4.51427219431353	-2.68312694389879
C	-0.20212249676145	-3.92809233560526	-0.39512661341362
C	3.82306088692724	-1.51919163365008	-1.88400714180539
H	4.31717567027823	-2.30809969304466	-2.47294948158729
H	4.58814994764586	-0.77210437873257	-1.62613918955540
H	3.06865396359776	-1.03298427652809	-2.51830287475249
C	1.19343984532683	-5.01469381863921	-2.07878126650837
H	1.33370814702736	-5.80142772525569	-2.82345743533562
C	4.23290413349252	-2.69988156943448	0.33343927677458
H	3.77340332102015	-3.02542029249023	1.27571679678359
H	5.01950460091047	-1.96661370696311	0.57063206094658
H	4.70975758808371	-3.57388166407993	-0.13925196077951
C	3.53971490079488	2.26680862520787	-2.39147753749470
H	2.84394608640254	1.96908722507784	-3.18931277818140
H	4.34209031868444	1.51758335093436	-2.34183683049430
H	4.00232562322519	3.22496708482941	-2.67755263729806
C	-0.65784361187054	4.96074848661047	-1.03816227465895
H	-1.53536736973542	5.61307743550534	-1.04369943183297
C	0.37900515854117	5.19010146756018	-1.94885361731962
H	0.31832480859103	6.02166930297717	-2.65449621730330
C	2.23084597174941	4.96883717902074	4.46869009419667
C	1.80351381052118	0.01845101360862	6.48382666592121
H	1.61198647386673	-0.86668825523900	7.10621799649151
H	1.59765847512877	0.90250046497359	7.10315335998940
H	2.86571569221240	0.02639422734074	6.20336425057220
C	-1.51484629629494	-3.83919723881118	0.37199255510588
H	-1.40914122605608	-3.01172605590260	1.08720174716866
C	2.57069693748984	4.96457350025389	5.97269312001489

H	1.68517312849944	4.73720268947377	6.58439302498194
H	2.94588684029127	5.95542355252244	6.27230246919392
H	3.34885191460898	4.22332159932865	6.20630055052584
C	3.60236208817117	-5.29737392739160	3.67746576603748
H	3.39084372772223	-5.37569527565716	2.60243042006518
H	4.00656274634577	-6.26447331793841	4.01869901516335
H	4.37780698430940	-4.52925227725456	3.81140600624707
C	2.68977641434655	-4.90783030432617	5.97488174957235
H	3.46608207570878	-4.15917557910606	6.19061653802158
H	3.07535776118921	-5.89369258653008	6.27774972847519
H	1.81178784895866	-4.68115146067673	6.59746569195799
C	-1.76695383069370	3.62829069348021	0.81604750428824
H	-1.49062494201309	2.76777950565374	1.43949711329569
C	3.80348695935514	2.78122450248427	0.07800710385997
H	4.19800825274332	3.79175466967853	-0.11581443399190
H	4.65463730664161	2.08391679831259	0.12686441768785
H	3.31062954611591	2.78851416699188	1.05816650019606
C	-3.03027450518260	3.23288648202494	0.02388212956140
H	-3.35205101686517	4.04413495073109	-0.64836897729934
H	-3.86116858484375	3.00859155258652	0.71078729037828
H	-2.83827239966264	2.33710881058847	-0.58335355616467
C	1.16165451869019	6.06022870209153	4.21831095665297
H	0.92106196766854	6.14497525006615	3.14984683581585
H	1.52056521398764	7.04271294588795	4.56607388852865
H	0.23197566033947	5.81982880804159	4.75434178576392
C	1.25275457729725	-6.01940240375242	4.25248641019254
H	0.33350839760624	-5.77387250877706	4.80399495649422
H	1.61598656074888	-7.00087329757039	4.59884763111417
H	0.99249049780048	-6.10772340920714	3.18896745941531
C	-2.68261988444805	-3.48362768047916	-0.56992880289122
H	-2.48951546939579	-2.52568413329382	-1.07303878771985

H -3.62211352369283 -3.38849427003325 -0.00346780475133

H -2.82592366264787 -4.25580666159364 -1.34260037981065

C 3.51786140394981 5.32685237158485 3.68426575568685

H 4.29255570013887 4.56363009713098 3.84753617979791

H 3.91395242191062 6.30187008008505 4.01248162136890

H 3.32395401041055 5.38041838603762 2.60435039892365

C -1.80181422224492 -5.12475400765287 1.17215724836153

H -1.91740195045682 -5.99711491833590 0.50924620962167

H -2.73169282745920 -5.01777759644211 1.75185845646611

H -0.98468811691835 -5.33396643603388 1.87574641109926

C -2.04059042995159 4.82299277257817 1.75046821199420

H -1.14751224270483 5.06665601651539 2.34023555763987

H -2.85672577773740 4.58401817620272 2.44960970256702

H -2.33597951149261 5.71957790461652 1.18205306519560

2-Pb:

Pb -0.13920403700416 -0.00878978581984 -0.23275304826141

O 0.36449578629254 0.00215090449684 2.42416339743191

N 0.61836774126693 2.04209318620813 0.76656895316153

N 0.62263494419484 -2.03564779295706 0.77914017482744

C 0.80819961201795 1.18439292975292 2.99248590814048

C 0.90094749885248 2.27064240450947 2.07976483915856

C 0.82874127062337 -1.17065599938253 2.99626909102968

C 2.05588718874850 -3.14238026082467 -0.87429328970622

C 1.18040032151944 -1.23509151400808 4.33775554307928

C 0.95071117276313 -2.25250158387575 2.08247748446186

C 0.93363221790379 0.00915086251240 5.20318268323965

C 1.58692054876252 3.33483344500730 -1.07639898936850

C 0.82918107316617 -3.06977067378737 -0.15455599151610

C 1.79386457821487 -3.58276092915498 3.98871085551919

C 1.60978633724702 2.49966873797104 4.83462303521870

H	1.88681392456357	2.57842351331564	5.88292747436239
C	1.15343428818261	1.25743301733513	4.33577948736053
C	1.69808175317144	3.62221331633104	3.99281083104321
C	0.54720536694301	3.13027577439578	-0.12255336517143
C	1.33384645620397	3.50188067518932	2.63701148755846
H	1.42035850983134	4.35850801855400	1.96634211028679
C	2.22595537921235	-4.16173711577846	-1.82594223551577
H	3.16692492087515	-4.22860393486677	-2.37742416469196
C	1.67076300339718	-2.46574888668559	4.83405487210453
H	1.94621044922723	-2.54022806203418	5.88314678206425
C	2.32146816817593	-4.93804463933629	4.49792266569403
C	3.17479646030052	-2.15209971373333	-0.57927557808760
H	2.71546515053832	-1.32355073865236	-0.01952580643728
C	1.43209497395203	-3.46858068891426	2.63164134533679
H	1.54075797167622	-4.32047881073569	1.95817879834103
C	-0.59200776024253	3.98468299786799	-0.11704496510314
C	-0.55596886693849	-0.00637461212998	5.65604533416091
H	-1.22169363559576	-0.01421041856738	4.78260482082046
H	-0.78116268022917	0.88830151811640	6.25638834349559
H	-0.76194022838486	-0.90481696460312	6.25766373636602
C	2.80823917813315	2.42323681170625	-1.05628962616282
H	2.43905647910182	1.41918440956504	-0.78698396326504
C	0.00407021164784	-5.01069315066610	-1.37729078797051
H	-0.78883445000566	-5.73590132089150	-1.58031999043109
C	1.47468186186943	4.40034548593149	-1.98518310935823
H	2.26558456609302	4.56683970456138	-2.71887081470482
C	-0.20615755988277	-4.00895690850813	-0.41368251511978
C	3.79452280241263	-1.56026785751859	-1.85706084345308
H	4.29042090209987	-2.32943968133192	-2.47019920456035
H	4.55190584159423	-0.80669054484623	-1.59452617261076
H	3.02325848111471	-1.07183634360082	-2.46928749749206

C	1.21222892919341	-5.09520678499197	-2.07898414963954
H	1.36269910214479	-5.88143279800948	-2.82216723201997
C	4.25149917256150	-2.78626558159441	0.32726788853063
H	3.80911018084409	-3.13836497919490	1.26813314804124
H	5.03224405303615	-2.04867936452513	0.57023501272862
H	4.73282883014895	-3.64286013020441	-0.17204630403188
C	3.51418740450132	2.31123035080516	-2.41631559303009
H	2.80986086391460	2.00949755734474	-3.20525133291264
H	4.31104675664698	1.55589011519200	-2.36432604108593
H	3.98164295208613	3.26233273278280	-2.71775638714243
C	-0.65691494356437	5.04415933725149	-1.03751336294101
H	-1.53009264766896	5.70252141874367	-1.03550252655670
C	0.36788443275674	5.25969791904398	-1.96576181262850
H	0.30211099353038	6.08583982593190	-2.67724204991186
C	2.21461019359146	4.98286420626880	4.49879858382467
C	1.83297592001481	0.01955900915306	6.45268481162161
H	1.64050183452390	-0.86616191032544	7.07379735219579
H	1.62315955466609	0.90211952497120	7.07264195448607
H	2.89628970999090	0.02962237329909	6.17647344233560
C	-1.52569782068730	-3.92259731561912	0.34158645637477
H	-1.43193888177971	-3.07971306054898	1.04086276878892
C	2.54094521914365	4.96238032931233	6.00567545997465
H	1.65130815981332	4.72171224093780	6.60628280049069
H	2.90720760288370	5.95175118287721	6.32098122245348
H	3.32149874514231	4.22309019341508	6.23730382574844
C	3.59364623178789	-5.32711054118268	3.70509491310510
H	3.38179228807388	-5.41623246801762	2.63090815839601
H	3.99094064233689	-6.29317340954850	4.05752852324875
H	4.37448182704315	-4.56306568822810	3.83072737925768
C	2.68160577200941	-4.90161249862596	5.99666627288361
H	3.46330953119693	-4.15611613014714	6.20359800190346

H	3.05908049428905	-5.88639071991562	6.31315810329706
H	1.80461333719129	-4.65975991610391	6.61496705135010
C	-1.74917671650626	3.73079630259017	0.84119989750368
H	-1.46051642500871	2.88443593865145	1.47825413918410
C	3.79918976807554	2.85451557729571	0.04595649117902
H	4.19506037777665	3.86176943166941	-0.16152264059892
H	4.64844833932453	2.15499282431939	0.09644998577884
H	3.31248194172093	2.87338135463855	1.02917323665064
C	-3.01787680418680	3.31455331043637	0.06817559633559
H	-3.34789709208789	4.11188221819543	-0.61654978703592
H	-3.84245561172112	3.09901947929538	0.76537536542552
H	-2.82868992944004	2.40840268761328	-0.52479963326266
C	1.14423985196486	6.07355426479983	4.25171481556598
H	0.91819400382522	6.17528800220015	3.18157194868186
H	1.49420055811118	7.05179284359664	4.62003311126470
H	0.20828800761056	5.82077952349354	4.77085758502912
C	1.23869110048419	-6.02483643139579	4.28681348964399
H	0.31993299986344	-5.76481442747419	4.83250110066671
H	1.59360124070086	-7.00424415703814	4.64749457204798
H	0.98036130143747	-6.12590882944148	3.22396147429939
C	-2.69518431718287	-3.60476341293656	-0.61150965066251
H	-2.51675989882573	-2.65639604366554	-1.13813438903652
H	-3.63810748097215	-3.50992340710866	-0.05087144315717
H	-2.82582154206771	-4.39579807003184	-1.36714303971775
C	3.50699620866095	5.35443614458742	3.72962849745635
H	4.28371765584267	4.59332529515772	3.89322598042581
H	3.89596980380241	6.32846837038010	4.06946472419026
H	3.32193535232801	5.41592065124016	2.64850291724793
C	-1.79924437003940	-5.19656330205034	1.16487348078022
H	-1.89878704939379	-6.08207414253787	0.51680164919170
H	-2.73343259653863	-5.09232390442568	1.73825787009619

H	-0.98260448562437	-5.38150105252539	1.87559941972275
C	-2.02276249516900	4.93999680216317	1.75618119577589
H	-1.12601202882655	5.19944235724483	2.33313980664309
H	-2.83060348499707	4.70811801125359	2.46727934316585
H	-2.32956177791261	5.82461738014565	1.17523205710608

S8) References

[S1] C. A. Cruz, D. J. H. Emslie, L. E. Harrington, J. F. Britten, C. M. Robertson, *Organometallics* **2007**, *26*, 692-701.

[S2] F. Krämer, M. S. Luff, U. Radius, F. Weigend, F. Breher, *Eur. J. Inorg. Chem*. **2021**, *35*, 3591-3600.

[S3] M. Solar, N. Trapp, mCHILL: *J. Appl. Cryst.* **2018**, *51*, 541-548.

[S4] J.; Cosier, A. M. Glazer, *J. Appl. Cryst.* **1986**, *19*, 105-107.

[S5] CrysAlisPro, Agilent Technologies, Version 1.171.35.8.

[S6] G. M. Sheldrick; *Acta Crystallogr.* **2015**, *A71*, 3-8.

[S7] G. M. Sheldrick, *Acta Crystallogr.* **2015**, *C71*, 3-8.

[S8] O. V. Dolomanov, L. J. Bourhis, R. J. Gildea, J. A. K. Howard, H. Puschmann, *J. Appl. Cryst.* **2009**, *42*, 339-341.

[S9] F. Neese, F. Wennmohs, U. Becker, C. Riplinger, *J. Chem. Phys.* **2020**, *152*, 224108.

[S10] E. Caldeweyher, S. Ehlert, A. Hansen, H. Neugebauer, S. Spicher, C. Bannwarth, S. Grimme, *J. Chem. Phys.* **2019**, *150*, 154122.

[S11] F. Neese. *Coord. Chem. Rev.* **2009**, *253*, 526-563.

[S12] D. A. Pantazis, F. Neese, *WIREs Comput. Mol. Sci.* **2014**, *4*, 363-374.

[S13] E. van Lenthe, E. J. Baerends, J. G. Snijders, *J. Chem. Phys.* **1993**, *99*, 4597-4610.

[S14] E. van Lenthe, E. J. Baerends, J. G Snijders, *J. Chem. Phys.* **1994**, *101*, 9783-9792.

[S15] C. van Wüllen, *J. Chem. Phys.* **1998**, *109*, 392-399.

[S16] J. D. Rolfes, F. Neese, D. A. Pantazis, *J. Comput. Chem.* **2020**, *41*, 1842-1849.

[S17] D. A. Pantazis, F. Neese, *Theor. Chem. Acc.* **2012**, *131*, 1292.

[S18] D. A. Pantazis, X. Y. Chen, C. R. Landis, F. Neese, *J. Chem. Theory Comput.* **2008**, *4*, 908-919.

[S19] F. Weigend, R. Ahlrichs, *Phys. Chem. Chem. Phys.* **2005**, *7*, 3297-3305.

[S20] B. A. Hess, C. M. Marian, U. Wahlgren, O. Gropen, *Chem. Phys. Lett.* **1996**, *251*, 365-371.

[S21] A. Berning, M. Schweizer, H. J. Werner, P. J. Knowles, P. Palmieri, *Mol. Phys.* **2000**, *98*, 1823-1833.

[S22] F. Neese, *J. Chem. Phys.* **2005**, *122*, 034107.

[S23] J. R. Morton, K. F. Preston, *J. Mag. Res.* **1978**, *30*, 577-582.

[S24] W. D. Woodul, E. Carter, R. Müller, A. F. Richards, A. Stasch, M. Kaupp, D. M. Murphy, M. Driess, C. Jones, *J. Am. Chem. Soc.* **2011**, *133*, 10074-10077.

[S25] S. A. Dikanov, Y. D. Tsvetkov, M. K. Bowman, A. V. Astashkin, *Chem. Phys. Lett.* **1982**, *90*, 149–153.

[S26] H. Flanagan, D. J. Singel, *J. Chem. Phys.* **1987**, *87*, 5606-561.

[S27] See for example a) Y.-C. Fann, N. C. Gerber, P. A. Osmulski, L. P. Hager, S. G. Sligar, B. M. Hoffman, *J. Am. Chem. Soc.* **1994**, *116*, 5989-5990; b) A. Veselov, H. Sun, A. Sienkiewicz, H. Taylor, R. M. Burger, C. P. Scholes, *J. Am. Chem. Soc.* **1995**, *117*, 7508–7512.

[S28] M. P. Egorov, O. M. Nefedov, T.-S. Lin, P. Gasper, *Organometallics* **1995**, *14*, 1539–1541.

[S29] J. D. Cotton, C. S. Cundy, D. H. Harris, A. Hudson, M. F. Lappert, P. W. Lednor, *J. C. S. Chem. Comm.* **1974**, *16*, 651-652.

[S30] A.; Sekiguchi, T. Fukawa, M. Nakamoto, V. Y. Lee, M. Ichinohe, *J. Am. Chem. Soc.* **2002**, *124*, 9865-9869.

[S31] T. Y. Lai, J. C. Fettinger, P. P. Power, *J. Am. Chem. Soc.* **2018**, *140*, 5674–5677.

[S32] T. Y. Lai, L. Tao, R. D. Britt, P. P. Power, *J. Am. Chem. Soc.* **2019**, *141*, 12527-12530.

[S33] A. Sekiguchi, T. Fukawa, V. Y. Lee, M. Nakamoto, *J. Am. Chem. Soc.* **2003**, *125*, 9250-9251.

[S34] M. Becker, C. Förster, C. Franzen, J. Hartrath, E. Kirsten, J. Knuth, K. W. Klinkhammer, A. Sharma, D. Hinderberger, *Inorg. Chem.* **2008**, *47*, 9965-9978.

[S35] D. Kurzbach, S. Yao, D. Hinderberger, K. W. Klinkhammer, *Dalton Trans.* **2010**, *39*, 6449-6459.

[S36] J. E. Bennett, J. A. Howard, *Chem. Phys. Lett.* **1972**, *15*, 322-324.
